# CDK12 orchestrates super‐enhancer‐associated CCDC137 transcription to direct hepatic metastasis in colorectal cancer

**DOI:** 10.1002/ctm2.1087

**Published:** 2022-10-17

**Authors:** Wei Dai, Junhong Wu, Xiaopeng Peng, Wen Hou, Hao Huang, Qilai Cheng, Zhiping Liu, Walter Luyten, Liliane Schoofs, Jingfeng Zhou, Shenglan Liu

**Affiliations:** ^1^ School of Pharmacy Gannan Medical University Ganzhou Jiangxi China; ^2^ Center for Immunology Gannan Medical University Ganzhou Jiangxi China; ^3^ Department of Biology KU Leuven Leuven Belgium; ^4^ Department of Hematology and Oncology International Cancer Center Shenzhen Key Laboratory Shenzhen University General Hospital Shenzhen University Clinical Medical Academy Shenzhen University Health Science Center Shenzhen China

**Keywords:** CCDC137, CDK12, colorectal cancer, liver metastasis, super‐enhancers

## Abstract

**Background:**

Hepatic metastasis is the primary and direct cause of death in individuals with colorectal cancer (CRC) attribute to lack of effective therapeutic targets. The present study aimed to identify potential druggable candidate targets for patients with liver metastatic CRC.

**Methods:**

The transcriptional profiles of super‐enhancers (SEs) in primary and liver metastatic CRC were evaluated in publicly accessible CRC datasets. Immunohistochemistry of human CRC tissues was conducted to determine the expression level of CDK12. Cellular proliferation, survival and stemness were examined upon CDK12 inhibition by shCDK12 or a selective CDK12 inhibitor named SR‐4835 with multiple in vitro and in vivo assays. RNA sequencing and bioinformatics analyses were carried out to investigate the mechanisms of CDK12 inhibition in CRC cells.

**Results:**

We identified CDK12 as a driver gene for direct hepatic metastasis in CRC. Suppression of CDK12 led to robust inhibition of proliferation, survival and stemness. Mechanistically, CDK12 intervention preferentially repressed the transcription of SE‐associated genes. Integration of the SE landscape and RNA sequencing, *BCL2L1* and *CCDC137* were identified as SE‐associated oncogenic genes to strengthen the abilities of cellular survival, proliferation and stemness, eventually increasing liver metastasis of CRC.

**Conclusions:**

Our data highlight the potential of CDK12 and SE‐associated oncogenic transcripts as therapeutic targets for patients with liver metastatic CRC.

## Graphical abstract

- Super‐enhancers fostered metastasis‐related cellular features (proliferation, survival, stemness) in CRC cells via driving overexpression of super enhancer‐associate genes *BCL2L1* and *CCDC137*.
- Disruption of SEs by CDK12 inhibition with SR4835 or shRNA lentivirus efficiently eliminated liver metastasis through downregulating transcription of relevant genes.
- CCDC137 promotes proliferation and cancer stem‐like cells in CRC.

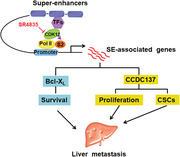

## 1 BACKGROUND

Successful targeting and eradication of metastatic tumour cells is one of the greatest challenges in patients with solid tumours.^1^ Indeed, almost 90% of cancer patients die from metastasis.^2^ The complicated processes of metastasis are principally composed of migration, invasion, intravasation and extravasation of blood vessel walls, circulation in the blood or lymphatic stream, colonisation, and proliferation in the host organs.^3^ During metastasis cascades, tumour cell migration and invasion are the earlier events. Cancer cells then inwardly traverse across blood or lymphatic vessels and become circulating tumour cells (CTCs). While only a minimum part of CTCs acquire abilities of stemness and apoptosis resistance, they can survive, colonise and proliferate in distant organs.^4^ Therefore, cellular features such as proliferation, survival and stemness are favourable for tumour cells to form metastatic lesions in distant organs.

Epigenetic alternations are prevalent in metastatic tumour cells, which endow cells to gain strengthened cellular features to colonise and proliferate in the host organs.^5^ Super‐enhancers (SEs), as a vital epigenetic regulation way, are extremely large clusters of enhancers that interact with promoters to enhance gene transcription.^6^ SEs recruit an extreme density of transcription factors/cofactors, cyclin‐dependent kinases (e.g., CDK7 and CDK12) and epigenetic regulators to synergistically drive transcriptional activity, which play a critical role in cell identity, proliferation, survival, etc.^6^ SEs are also acquired by tumour cells around key oncogenes, such as *XBP1*,^7^ *PAK4*,^8^ *MYCN*.^9^ It has been reported that interruption of SEs can selectively inhibit transcription of the associated oncogenes and induce regression in many types of cancers including melanoma,^10^ hepatocellular carcinoma^11^ and small cell lung cancer.^12^ However, whether SEs and their associated oncogenes are involved in liver metastasis in colorectal cancer (CRC) remains unknown.

CDK12 forms a heterodimeric complex with cyclin K, which plays critical roles in regulating transcriptional elongation by phosphorylation of serine 2 of the RNA polymerase II (Pol II) C‐terminal domain (CTD).^13^ Previous studies demonstrated that CDK12/13 inhibition preferentially decreases the expression of key SE‐associated genes and DNA damage response genes in tumour cells.^14^, ^15^ Moreover, inhibition of CDK12 is striking lethal in Ewing sarcoma breakpoint region 1 protein‐Friend leukaemia integration 1 transcription factor oncogene‐positive Ewing sarcoma.^16^ Furthermore, targeting CDK12 exhibited a significant decrease in the cellular proliferation of liver cancer via induction of DNA damage response^17^ and impaired osteosarcoma cell colonisation by downregulation of MYC proto‐oncogene (MYC).^1^ Finally, transcriptional repression by targeting CDK12 has been suggested as a encouraging strategy to impede the progresssion of cancers with no definitive drivers, such as triple negative breast cancer (TNBC)^15^ and osteosarcoma.^1^ A recent study has reported that CDK12 knockout decreases proliferation of TP53 deficiency CRC cells.^18^ However, the role of CDK12 and its mechanism in CRC with unknown drivers of liver metastasis are not well defined.

In this study, we found that the SE landscape in CRC was implicated in liver metastasis. Concomitantly, CDK12, a critical component of the SE complex, was overexpressed in CRC cell sublines with high liver metastatic competence and in patients with liver metastatic CRC. Furthermore, metastasis‐associated cellular features including proliferation, survival and cancer stem cells (CSCs) of CRC cells were greatly dependent on transcription of SE‐associated oncogenes (e.g., *BCL2L1*, *CCDC137*), which were exceptionally vulnerable to SE interruption by CDK12 inhibition. Our studies may shed light on the understanding of the mechanism that underlies liver metastasis, providing potential therapeutic targets for hepatic metastatic CRC treatment.

## 2 MATERIALS AND METHODS

### 2.1 Materials and antibodies

SR‐4835, olaparib and BMS‐202 were obtained from TargetMol (Shanghai, China), and prepared as a stock solution with a concentration of 20 mM and kept in aliquots at –20°C. Propidium iodide (PI) and Annexin V‐FITC were bought from Sigma–Aldrich (Shanghai, China). The following antibodies were used for Western blotting: CDK12 (Cell Signaling Technology: CST, 11973), CDK13 (BOSTER, A05292‐1), RNA Pol II CTD Ser2 (Bethyl, A300‐654A), total RNA Pol II (Bethyl, A300‐653A), RNA Pol II CTD Ser5 (Bethyl, A304‐408A), Poly ADP‐Ribose Polymerase (PARP) (CST, 9532), Caspase‐3 (CST, 9662), Survivin (Proteintech, 10508‐1‐AP), active caspase‐3 (CST, 9661), Bcl‐X_L_ (Santa, SC‐634), Bcl‐2 (Millipore, 05–729), Mcl‐1 (Proteintech, 16225‐1‐AP), γH2AX (CST, 9718S), CCDC137 (Abcam, ab185368) and β‐actin (Sigma–Aldrich, A1978).

### 2.2 Colony‐formation assay

The colony‐formation ability of CRC was assessed with drug‐free double layer soft agar system as previously described.^19^ In brief, approximately 10, 000 CRC cells were suspended with a top layer containing 0.5% agar (Invitrogen, Guangzhou, China) in Dulbecco's Modified Eagle's Medium (DMEM) or PRMI 1640 containing 10% fetal bovine serum (FBS), and then cultured on the bottom layer of 10% FBS in the 24‐well plates with solidified 1% agar in DMEM or PRMI 1640. After 1 week, the colonies that consisted of more than 50 cells were counted via using a microscope.

### 2.3 Tumoursphere formation

Tumoursphere formation assay was conducted according to as previous study.^20^ Briefly, after exposure to SR‐4835 for 24 h, CRC cells were harvested and resuspended with DMEM/F12 medium containing with 2% B27, 20 ng/ml epidermal growth factor and 10 ng/ml basic fibroblast growth factor, and then seeded into a low attachment 24‐well plate (ThermoFisher, Shanghai, China). Seven days later, tumourspheres with more than 50 cells were calculated. The tumourspheres were then harvested, softly separated into a single cell and re‐plated with the same density as the primary round (5000 cells/well) for the secondary and tertiary rounds of culture.

### 2.4 Quantitative real‐time polymerase chain reaction assay

Quantitative real‐time polymerase chain reaction (qRT‐PCR) assay was performed according to the manufacturer's instructions. Trizol (Invitrogen) was employed to obtain total RNA from CRC cells or tissues. RNA was then reversely transcribed to cDNA with One‐step RT Kit (ThermoFisher) in accordance with the manufacturer's instruction. The qRT‐PCR reaction was performed on a BioRAD Real‐Time PCR System. Gene expression was standardised to the expression of GAPDH with the $2^{-\Delta \Delta C_{T}}$ approach, and ΔΔ*C*_T_ = (*C*_T, Target gene_ – *C*_T, GAPDH_)_treated cells_ –  (*C*_T, Target gene_ – *C*_T, GAPDH_)_control cells_. The qRT‐PCR primers are presented in Table S1.

### 2.5 Transfection experiments

Guide RNAs (gRNAs) were cloned into the lenti‐sgRNA‐puro or lenti‐sgRNA‐MS2‐Zeo (msgRNA) backbone plasmids. For gene activation experiments, CRISPR activation (CRISPRa) assay with synergistic activation mediator (SAM) complex was employed.^21^ In brief, HCT116 cells were transfected with dCas9‐VPR (MiaoLing, P10265, Guangzhou, China) and specific individual msgRNA or nontargeting Scramble msgRNA at a ratio of 2:1. For gene repression experiments, CRISPR inhibition (CRISPRi) assay with dCas9‐KRAB repressor complex was employed.^22^ HCT116 cells were transfected with dCas9‐KRAB‐MeCP2 (Addgene, 110824, MA, USA) and specific individual sgRNA or nontargeting Scramble sgRNA at a ratio of 2:1. Forty‐eight hours after transfection, cells were collected for qRT‐PCR and Western blotting assay. After verification of transfection efficiency, cells will be used for proliferation, migration and invasion experiments with CCK8 and transwell assays, respectively. The detailed target sequences of gRNAs are provided in Table S2.

### 2.6 Generation of stable cell lines

The lentivirus was produced by transfection with the constructs [pLKO.1‐puro‐non‐target Vector (Scramble), pLKO.1‐puro‐shCDK12, pLKO.1‐puro‐shCDK13, pLKO.1‐puro‐shBCL2L1, pLKO.1‐puro‐shCCDC137, pTSB‐puro‐Vector, pTSB‐puro‐BCL2L1 or pTSB‐puro‐CCDC137 from Transheep (Shanghai, China)] in 293T cells using the lentivirus packing system [the pCMV‐VSVG (envelope construct), pCMV‐dR8.2 (the packing construct)]. After transfection for 48 and 72 h, viral supernatants were collected and filtered utilising the 0.45 μM filters. CRC cells were infected with viral supernatants containing 8 μg/ml polybrene (Sigma–Aldrich) for two rounds, and then subjected to puromycin (1 μg/ml) treatment for approximately 5 days to select stable clones. For establishment of stable overexpression of CDK12, CRC cells were transfected with pcDNA3.1‐puro‐Vector and pcDNA3.1‐puro‐CDK12 plasmid using polyethyleneimine (Polysciences, Inc., Warrington, PA, USA). The transfected cells were then incubated with puromycin (1 μg/ml) for about 5 days to select stable clones. The detailed sequences of shRNA are listed in Table S3.

### 2.7 RNA sequencing

After HCT116 cells were incubated with SR‐4835 for 6 h or stable CDK12‐depledted cells were established, Rneasy Mini Kit (Qiagen) was utilised to isolate RNA. Sequencing libraries were constructed by NEBNext UltraTMRNA Library Prep Kit for Illumina (NEB, USA) according to the manufacturer's recommendations. Genes with mean expression of fragments per kilobase of transcript per million mapped reads (FPKM) >1 were defined as active transcripts, which used for subsequent analysis. The heatmap was analysed with TBtools and the boxplot was drawn using BoxPlotR (http://shiny.chemgrid.org/boxplotr/), separately.

### 2.8 Microarray data analysis

For published microarray datasets, SEs identified in HCT116 and SW620 cells were downloaded from SEdb^23^ and dbCoRC^24^ database, respectively. GSE49355, GSE50760, GSE42158 and GSE21510 were downloaded from Gene Expression Omnibus (GEO) database. Additionally, we obtained the gene expression from The Cancer Genome Atlas Program (TCGA) database.

### 2.9 SE definition

ROSE software (https://bitbucket.org/young_computation/rose) was utilised to identify SEs according to previous studies.^7^, ^8^ Briefly, based on the signal of Chromatin immunoprecipitation‐sequencing (ChIP‐Seq) with H3K27ac antibody, the stitched peaks within 12.5 kb were ranked and plotted. SEs were then designated as the signal of peaks that above the inflection point. SEs and typical‐enhancers (TEs) were assigned to the closest genes.

### 2.10 ChIP‐qPCR

ChIP‐qPCR assay was conducted as previously described.^19^, ^25^ Briefly, 1 × 10^7^ SR‐4835‐treated or control CRC cells were cross‐linked with 1% formaldehyde. Cells were then harvested to break into 200–500 bp DNA fragments using a sonicator. The following antibodies were used for immunoprecipitation with chromatin complexes: normal immunoglobulin G (IgG) (CST, 2729S), anti‐CDK12 (CST, 11973) or anti‐RNA Pol II (Bethyl, A300‐653A). To digest RNA and protein in the protein–DNA complexes, RNase A and proteinase K were employed, respectively. The qPCR reaction was adopted to measure the precipitated DNA fragments. The specific primers used for ChIP‐qPCR were provided in Table S4.

### 2.11 Gene Ontology and gene set enrichment analysis

DAVID tool V6.8 (https://david.ncifcrf.gov/) was used for Gene Ontology (GO) analysis. Sensitive transcripts were considered as assayed gene, which decreased ≥1.5‐fold upon SR‐4835 treatment for 6 h or CDK12 depletion in HCT116 cells. GO analysis was also used for SE‐associated genes. Gene set enrichment analysis (GSEA) standalone desktop program was used for GSEA analysis. The expression matrix was included transcripts in primary CRC patients and liver metastatic CRC patients or expression values with exposure to 100 nM of SR‐4835 for 6 h; and gene set included SE‐associated genes identified in CRC cells.

### 2.12 Human CRC tissue samples

Under informed consent from each subject or each subject's guardian following the institutional policies and the Declaration of Helsinki principles, a total of 66 tissues with CRC (15 adjacent noncancerous tissues, 30 primary tumours and 21 liver metastasis tumours) from different individuals were collected at GanZhou Cancer Hospital from 2016 to 2021 and subjected to immunohistochemistry (IHC) assays. Eighteen fresh pairs of normal colon tissues, primary and liver metastatic CRC tissues were collected from operated patients at Gannan Medical University's First Affiliated Hospital and subjected to qRT‐PCR or Western blotting assay. All these samples were diagnosed based on histological examinations.

### 2.13 Immunohistochemistry

The expression of CDK12 and CCDC137 in CRC patient tissues were detected by IHC assay, respectively. Sample slides with 4 μm thick were incubated with xylene, rehydrated using ethanol and antigen retrieval. IHC were then performed using antibody against CDK12 or CCDC137 at a ratio of 1:100 dilution. Sections were scored according to previous study.^19^ In brief, the staining intensity was scored as followed: no staining received a score of 0; faint staining a score of 1; moderate staining a score of 2; and intense staining a score of 3. The ratio of positive‐staining cells was scored 0, 0%; 1, <10%; 2, 10%–40%; 3, 41%–70%; 4, >70%. For each sample, the staining intensity score and the ratio of the positive cells value were multiplied, resulting in scores ranking from 0 to 12.

### 2.14 Xenograft experiment

HCT116 cells (5 × 10^6^ per mouse) were subcutaneously implanted into the flanks of male BALB/c nude mice aged 4–6 weeks (GemPharmatech, Jiangsu, China) to establish xenografts. Every other day, the volume of tumours were measured using the calipers and calculated by the formula: 0.4 × *a* × *b* ^2^, where *a* represents the long axis and *b* represents the short axis. When the tumours had grown to ∼50 mm^3^, mice were randomly divided into two groups with six mice per each group and given with SR‐4835 (20 mg/kg with 5 days on, 2 days off per week) or vehicle [10/90 dimethyl sulfoxide (DMSO)/30% hydroxypropyl‐b‐cyclodextrin in water] orally for 2 weeks. Tumours were harvested, photographed and weighed after mice were sacrificed. Part of tumour tissues were lysed for Western blotting assay, while the remaining tumour section was used for haematoxylin–eosin (H&E) and IHC staining.

### 2.15 Limiting dilution assay

After treatment with control (DMSO, less than 0.1%, v/v) or 100 nM SR‐4835 for 48 h, viable HCT116 cells were collected and inoculated subcutaneously in the flanks of male BALB/c nude mice aged 4–6 weeks (GemPharmatech) at successive concentrations (5 × 10^6^, 1 × 10^6^, 5 × 10^5^, 1 × 10^5^ per mice), five mice each group. When tumour volumes in control mice with 5 × 10^6^ cells reached ∼1500 mm^3^, the numbers of xenografts for each group were collected to record for CSC frequency calculation with L‐Calc dilution software (Stem Cell Technologies Inc.)

### 2.16 Liver metastatic mouse model

HCT116 cells (5 × 10^6^) stably expressing luciferases (HCT116‐luc) were injected into the spleen of male BALB/c nude mice aged 4–6 weeks (GemPharmatech). Ten minutes later, the spleen was removed to minimise the effect of splenic tumour. Quantification of photon flux in the liver zone is weekly measured by in vivo bioluminescence imaging (BLI) post‐injection of HCT116‐luc cells for 3 weeks. The livers of mice were resected at the end of the experiments, followed by Bouin's solution fixation for 1 week. After counting the metastatic nodules on liver surface, H&E staining was performed.

### 2.17 Orthotopic mouse metastatic model

The orthotopic mouse metastatic model was established as previous.^26^ In brief, 5 × 10^6^ HCT116 cells per mouse were subcutaneously inoculation into 4–6‐week male BALB/c nude mice (GemPharmatech). After 14 days, the mice were euthanised and the tumours were resected to cut into 2–3 mm diameter pieces. The CRC tumour pieces were surgically inserted into the caecum of anaesthetised nude mice. Approximately 2 months later, the mice were scarified and individual organs were isolated and treated with Bouin's solution. After calculating the metastatic nodules on liver surface, H&E staining was conducted. All animal researches have been approved by the Animal Experimental Ethics Committee of Gannan Medical University.

### 2.18 Statistical analysis

GraphPad Prism 5.0 Software (San Diego, CA, USA) was used for the statistical analysis. Two‐tailed Student's *t*‐test was employed for comparisons between two groups. One‐way analysis of variance, post hoc intergroup comparisons by Tukey's test was adopted for comparisons among multiple groups. Kaplan–Meier survival was analysed by log‐rank test. The data were shown as mean ± standard deviation. Statistics were considered significant at *p* <.05.

## 3 RESULTS

### 3.1 The super‐enhancer landscape in colorectal cancer is involved in liver metastasis

**FIGURE 1:**
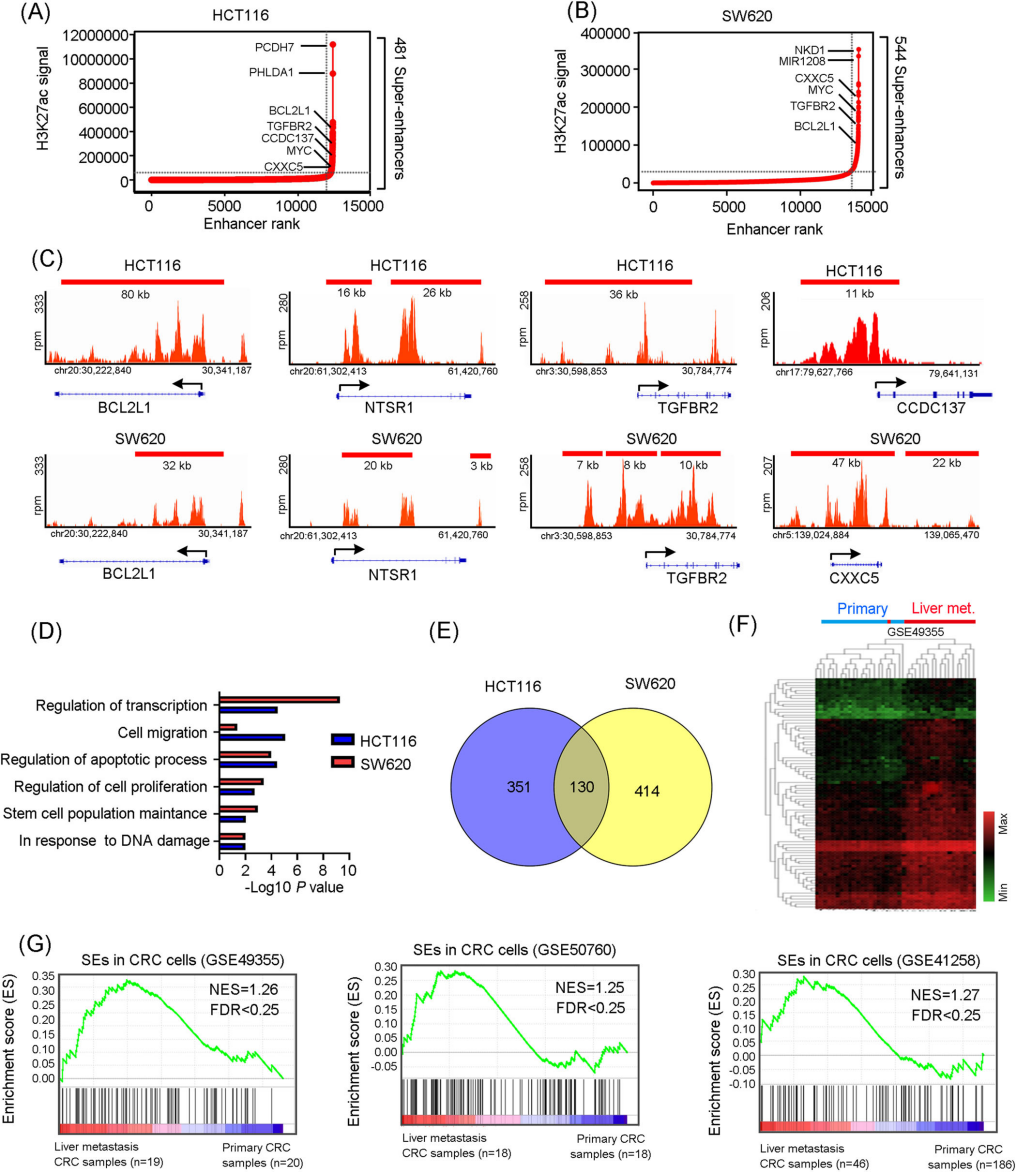
The super‐enhancer (SE) landscape in colorectal cancer (CRC) is correlated with liver metastasis. (A and B) Enhancer regions of HCT116 (A) and SW620 (B) cells were plotted based on increasing H3K27ac ChIP‐Seq signal obtained from SEdb and dbCoRC database, respectively. SEs were defined as the enhancers above the inflection point of the curve. (C) Representative H3K27ac ChIP‐Seq profiles at specific SE‐associated gene loci in HCT116 and SW620 cells. (D) Gene Oncology (GO) enrichment analysis of biological processes for SE‐associated genes in HCT116 and SW620 cells by DAVID 6.8. (E) One hundred and thirty SE‐associated genes were commonly shared in HCT116 and SW620 cells. (F) The expression profile of the shared SE‐associated genes in HCT116 and SW620 cells was clearly segregated between CRC primary samples and liver metastatic samples in the unsupervised clustering analysis. (G) Gene set enrichment analysis (GSEA) of commonly shared SE‐associated genes in HCT116 and SW620 cells were enriched in liver metastatic CRC tumours instead of primary CRC tumours in the GSE49355, GSE50760 and GSE41258 datasets.

To explore the implications of SEs in CRC, we generated a catalog of SEs in HCT116 and SW620 cells, two CRC cell lines, based on the publicly available SEdb and dbCoRC database. Four hundred and eighty‐one and 544 SE‐associated genes in HCT116 and SW620 cells were respectively identified (Figure 1A,B). Notably, most of the top‐ranked SE‐associated genes were well‐known oncogenes in CRC, such as *BCL2L1*,^27^ *LIF*,^28^ *NTSR1* ^29^ and *TGFBR2* ^30^ (Figure 1A,B). These observations suggest that SEs may implicate in driving transcription of multiple oncogenes in CRC cells. Moreover, we found several novel SE‐associated transcripts, including *CCDC137* and *CXXC5* (Figure 1C), whose functions have not been reported in CRC biology.

Next, we conducted GO analysis to investigate the biological functions of these SE‐associated genes. These genes were found to play a key role in cellular traits of tumour metastasise to distant organs, such as regulation of apoptosis, cell proliferation, stemness, migration and transcription (Figure 1D). Furthermore, 130 SE‐associated genes were commonly acquired in both HCT116 and SW620 cells, which were clearly segregated liver metastatic CRC samples from primary CRC samples (Figure 1E,F). Importantly, GSEA displayed that these commonly acquired SE‐associated genes were enriched in liver metastatic CRC samples instead of primary CRC samples in published GEO datasets (Figure 1G). Overall, these results suggest that CRC cells may acquire SEs to enhance the transcription of oncogenes to modulate cellular features (e.g., proliferation, survival and stemness), thereby promoting CRC cells metastasise to liver.

### 3.2 CDK12 is overexpressed in the highly liver metastatic CRC cell sublines and in liver metastatic CRC patients, which is also correlated with poor prognosis in CRC

High levels of epigenetic regulators, transcription factors, cofactors and cyclin‐dependent kinases were found to be recruited by SEs as the fuel to drive the active transcription of SE‐associated genes.^11^ Hence, we mainly focused on MED1, BRD4, EP300, CDK7, CDK12 and CDK13. MED1, as the critical component of the SE complex, is essential for gene transcription by RNA Pol II.^31^ BRD4, an important epigenetic regulator, drives oncogenic transcription by recognising acetylated lysine of histone and recruiting many other proteins to promoters.^32^ EP300, a critical component of the CBP/p300 complex, is the major histone acetyltransferase on SEs.^33^ CDK7, CDK12 and CDK13 function as transcriptional cyclin‐dependent kinases. For details, CDK7 phosphorylates RNA Pol II at serine 5 (S5) to regulate the initiation of transcription^8^ and CDK12/13 modulates transcriptional elongation by phosphorylating RNA Pol II at serine 2 (S2).^17^

**FIGURE 2:**
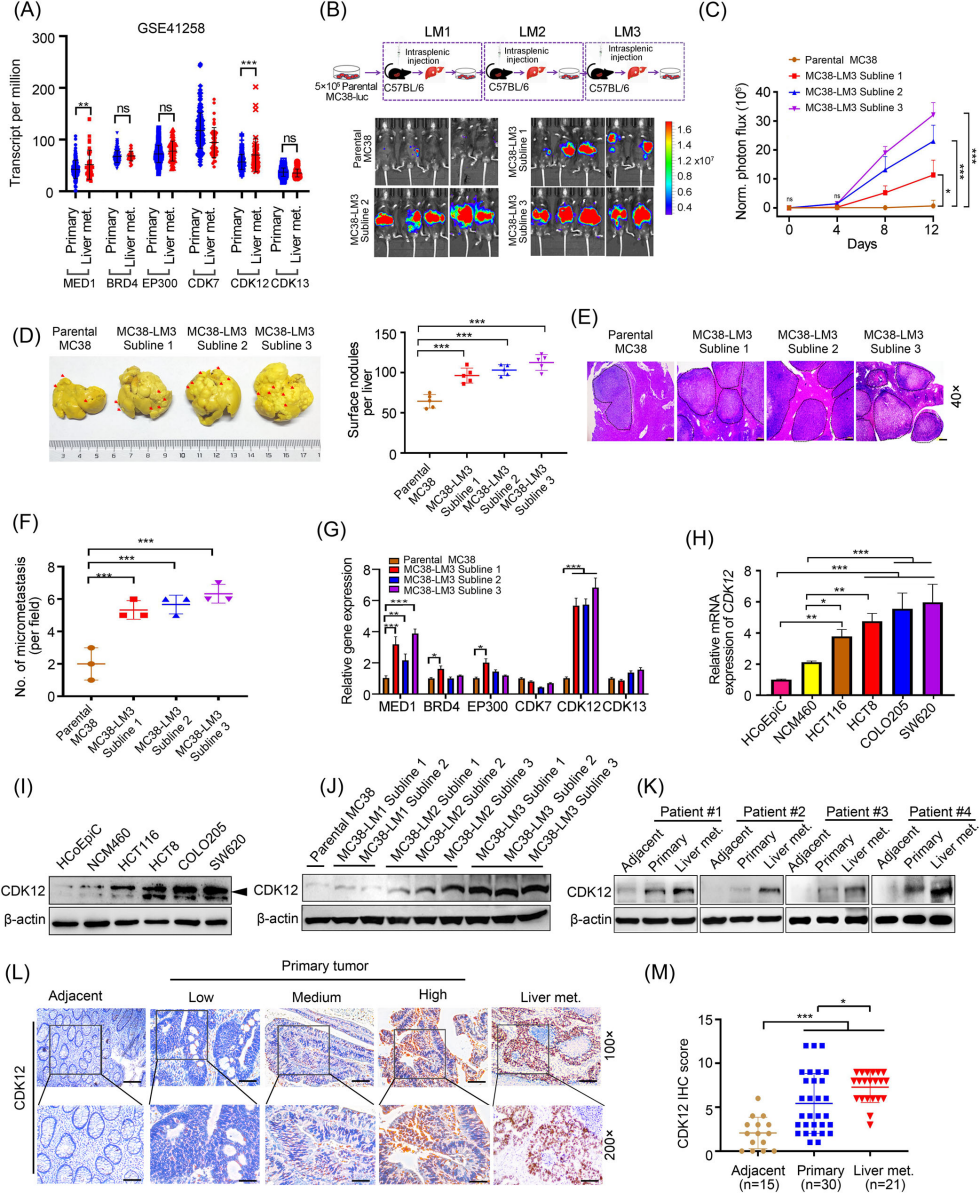
CDK12 is overexpressed in the highly hepatic metastatic colorectal cancer (CRC) cell sublines and in liver metastatic CRC patients, which is also correlated with poor prognosis in CRC. (A) The expression levels of super‐enhancer (SE) complex components, including MED1, BRD4, EP300, CDK7, CDK12 and CDK13 in primary CRC samples (*n* = 46) and liver metastatic CRC samples (*n* = 186) were shown. (B) Top: flowchart of in vivo‐selected highly liver metastatic cell derivatives in CRC was shown. MC38‐luc cells (5 × 10^5^) were intrasplenically inoculated into C57BL/6 mice for 12 days to isolate cell populations that spread to the liver. After single metastatic nodule on liver surface was dissociation and expansion in culture, the resulting cells (liver metastatic derivative 1, named LM1) were subjected to the secondary and tertiary round of in vivo selection, respectively, yielding the liver metastatic derivative 3 (LM3) cell populations. The highly liver metastatic subclones referred as MC38‐LM3 Subline 1, MC38‐LM3 Subline 2 and MC38‐LM3 Subline 3 were isolated from the liver metastatic foci of the third round in vivo experiments. Bottom: MC38‐LM3 subclones displayed a boosted capacity for metastasis to liver. The parental MC38 and MC38‐LM3 sublines were intrasplenically injected into C57BL/6 mice and bioluminescence imaging (BLI) photon flux was captured every week. Representative images of BLI on day 12 were shown. *n* = 5 per group. (C) Quantification of BLI photon flux for hepatic metastases in C57BL/6 mice was shown. (D) Representative photomicrograph of liver after Bouin's fixation (left) and quantification of liver surface metastatic nodules on day 12 after injection of 5 × 10^5^ CRC cells (right) were shown. Arrows indicated the distribution metastatic foci, *n* = 5 each group. (E and F) Representative images of haematoxylin–eosin (H&E) staining of liver section (E) and quantification of the number of liver metastatic foci in microscopic fields were displayed (F). *n* = 3 per group. Scale bar: 100 μm. (G) The mRNA levels of SE complex components including MED1, BRD4, EP300, CDK7, CDK12 and CDK13 in parental MC38 cells and aggressive LM3 subclones were analysed with quantitative real‐time polymerase chain reaction (qRT‐PCR). (H and I) The mRNA (H) and protein (I) levels of CDK12 in human normal colonic epithelial cells (HCoEpiC and NCM460) and CRC cells (HCT116, HCT8, COLO205 and SW620) were analysed with qRT‐PCR and Western blotting, respectively. (J) Expression levels of CDK12 in parental MC38 cells and aggressive MC38‐liver metastatic subclones were detected with Western blotting assay. (K) Expression levels of CDK12 in human primary CRC tumours, liver metastatic CRC tumours and adjacent normal tissue were examined by Western blotting. (L) Representative immunohistochemistry (IHC) images of CDK12 expression in human primary CRC samples, liver metastatic CRC samples and adjacent normal tissue. Scale bar: 200 μm (100×), 100 μm (200×). (M) The staining intensity of CDK12 was scored by multiply area score and light score. ns, not significant; ^**^ *p* < .01; ^***^ *p* < .001, Student's *t*‐test for result in (A). ns, not significant; ^*^ *p* < .05; ^**^ *p* < .01; ^***^ *p* < .001, one‐way analysis of variance (ANOVA) with post hoc intergroup comparison by Tukey's test for results in (C), (D), (F–H) and (M)

We found that the expression of CDK12 out of the six genes was topmost in liver metastatic CRC patients compared with primary CRC patients as revealed by GSE41258 dataset (Figure 2A). To further explore whether the components of the SE complex associated with CRC liver metastasis, we yielded MC38 cell sublines with highly liver metastatic competence through three rounds of intrasplenic injection of MC38 cells into C57 BL/6 mice according to previous study (Figure 2B).^34^ The sublines resulted from the third round of in vivo selection were named as liver metastatic derivative 3 (LM3). We then confirmed that MC38‐LM3 sublines (Subline #1, Subline #2 and Subline #3) significantly enhanced liver metastatic competence relative to the parental MC38 cells evidenced by luciferase intensity (Figure 2B,C), liver size and number of surface metastatic nodules (Figure 2D). Similarly, H&E staining examination revealed that much more metastatic foci were found on the livers of MC38‐LM3 sublines‐inoculated mice relative to parental MC38 cells‐treated mice (Figure 2E,F). Consistent with results in patients with liver metastatic CRC, the abundant levels of CDK12 were the highest compared with the other five components of SE complex in highly liver metastatic MC38‐LM3 sublines by qRT‐PCR analysis (Figure 2G). In addition, higher mRNA and protein levels of CDK12 were found in the CRC cells without metastatic capabilities (HCT116 and HCT8) and CRC cells with metastatic capabilities (COLO205 and SW620) relative to human normal colonic epithelial cells (NCM460 and HCoEpiC) (Figure 2H,I). Of importance, the expression of CDK12 was increased along with enhanced liver metastatic competence in MC38 cells as revealed by Western blotting (Figure 2J). By using Western blotting to further examine paired adjacent normal colon tissues, primary CRC tumours and liver metastatic CRC tumours, it was shown that CDK12 were boosted in adjacent normal colon tissues, primary CRC tissues and liver metastatic tissues in order (Figure 2K). IHC results confirmed that CDK12 was more abundant in CRC tissues than adjacent normal colon tissues, most abundant in the subjects with liver metastatic CRC (Figure 2L,M). These findings indicate that CDK12 may play a key role in facilitating liver metastasis of CRC. Therefore, in the subsequent experiments, CDK12 was the primary focus.

We next conducted analysis of TCGA database and the CPTAC database in UALCAN website. The result verified that CRC tissues had significantly higher levels of CDK12 protein and mRNA than those normal tissues (Figure S1A,B). Moreover, there was a strong correlation between CDK12 expression and lymph node metastatic stages in CRC (Figure S1C). Meanwhile, consistent with our results, GEO data also revealed that metastatic CRC patients exhibited a considerably higher expression of CDK12 compared to those non‐metastatic patients (Figure S1D,E). In addition, GSE41258 dataset further confirmed that CDK12 was orderly increased in adjacent normal colon tissues, primary CRC tissues and metastatic CRC samples (Figure S1F). Importantly, both the TCGA and GEO database indicated that the overexpressed CDK12 was positively correlated with short survival in CRC patients (Figure S1G,H). Collectively, these data imply that the expression of CDK12 is positively associated with liver metastasis and poor prognosis in CRC.

### 3.3 Global and selective transcription repression by targeting CDK12 in CRC cells

**FIGURE 3:**
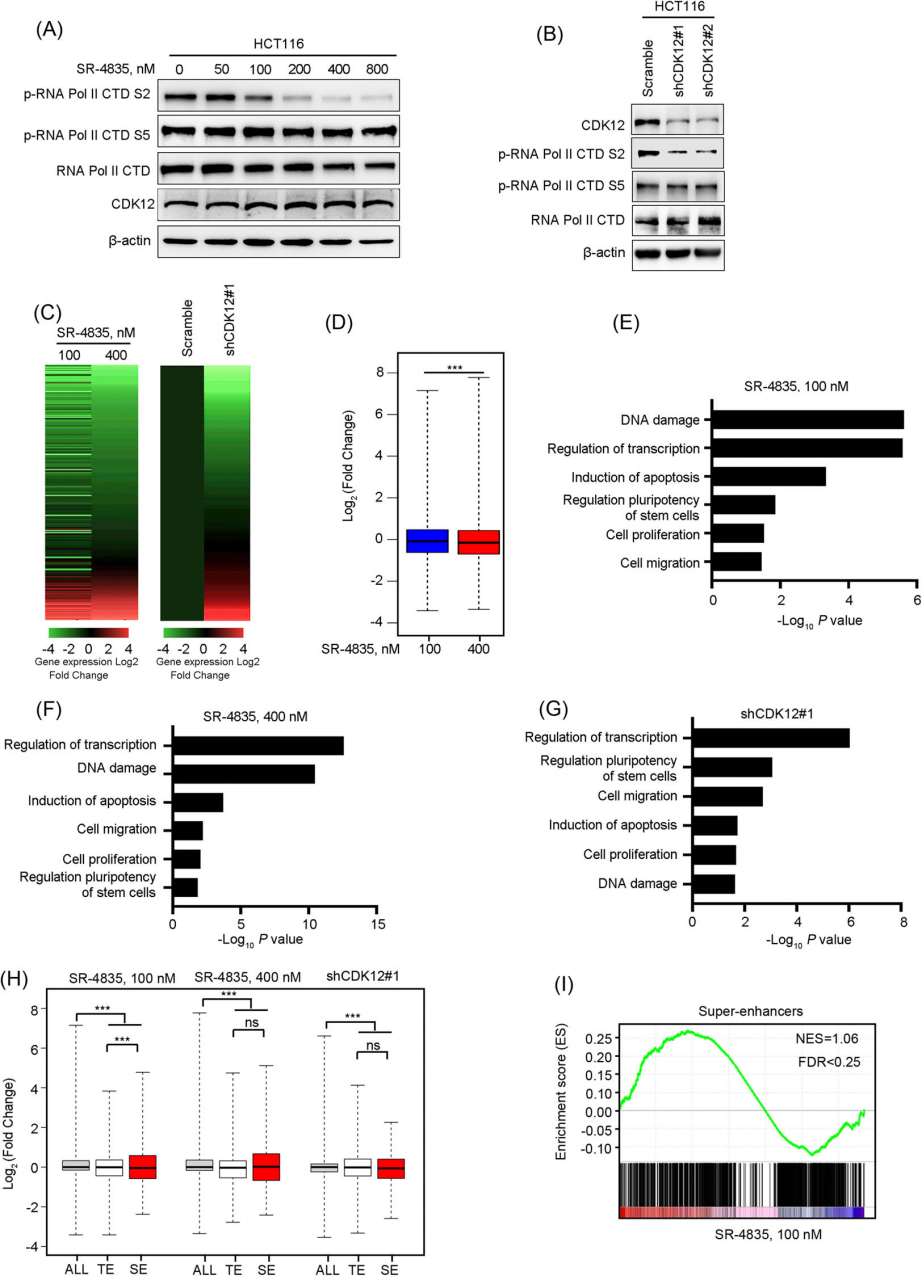
CDK12 inhibition disrupts RNA polymerase II (Pol II)‐enabled gene transcription in colorectal cancer (CRC) cells. (A) Western blotting analysis of RNA Pol II C‐terminal domain (CTD) phosphorylation in HCT116 cells after treated with increasing concentrations of SR‐4835 for 6 h was shown. (B) Western blotting analysis of RNA Pol II CTD phosphorylation in CDK12‐silenced HCT116 cells was shown. (C) Heatmaps of global gene expression in high concentration (400 nM) or low concentration (100 nM) SR‐4835‐treated HCT116 cells for 6 h (left) or in CDK12‐depleted HCT116 cells (right) were shown. (D) Box plots of log2 fold changes of global gene expression in high concentration (400 nM) or in low concentration (100 nM) SR‐4835‐treated HCT116 cells relative to dimethyl sulphoxide (DMSO)‐treated HCT116 cells for 6 h were presented. (E–G) Gene Oncology (GO) analysis of enriched biological processes of downregulated genes (≥1.5‐fold) in low concentration (100 nM) or in high concentration (400 nM) SR‐4835‐treated HCT116 cells (E and F) or in CDK12‐knockdown HCT116 cells (G) were shown. (H) Box plots of log2 fold changes for all transcripts (ALL), genes associated with typical‐enhancers (TEs) and super‐enhancers (SEs) in high concentration (400 nM) or in low concentration (100 nM) SR‐4835‐treated HCT116 cells for 6 h or in CDK12‐depleted HCT116 cells were shown. (I) Gene set enrichment analysis (GSEA) of SE‐associated genes in low concentration (100 nM) of SR‐4835‐treated HCT116 cells were shown. ^***^ *p* < .001; two‐sided Mann–Whitney *U*‐test for results in (D) and (H)

CDK12 regulates transcriptional elongation processing by phosphorylating the RNA Pol II CTD at S2. In keeping with this role, we found that CDK12 inhibition by the compound SR‐4835 for 6 h or specific shRNA led to a evident decrease in S2 phosphorylation of RNA Pol II CTD with a minimal effect on the transcription initiation‐associated S5 in CRC cells (Figure 3A,B). We next investigated the impact of CDK12 inhibition on gene expression profile by executing RNA sequencing (RNA‐Seq) assay. The results showed that a high concentration of SR‐4835 (400 nM) or CDK12 knockdown with almost complete inhibition of the phosphorylation of S2 in RNA Pol II CTD resulted in a global decline of mRNA levels, while a low concentration of SR‐4835 (100 nM) with partially blocking the phosphorylation of RNA Pol II CTD S2 led to selective downregulation of a group of transcripts (Figure 3C,D). GO analysis of the sensitive genes (reduced ≥1.5‐fold change with SR‐4835 treatment or CDK12 depletion) was enriched in processes of transcriptional regulation, DNA damage, stem cell division, apoptosis, cell proliferation and cell migration in CRC cells, which are essential for cancer metastasis (Figure 3E–G). Moreover, we found that both TE‐ and SE‐associated genes were significantly downregulated upon high concentration of SR‐4835 treatment or depletion of CDK12 (Figure 3H). However, compared with TE‐associated genes, the expression levels of SE‐associated genes were decreased to a higher degree after low concentration of SR‐4835 treatment, suggesting SE‐associated genes were especially vulnerable to partially suppressing transcription (Figure 3H). The GSEA result further confirmed that SE‐associated transcripts were enriched in low dose of SR‐4835‐treated (100 nM) cells compared with DMSO‐treated cells (Figure 3I). These data indicate that the mRNA levels of SE‐associated genes are particularly vulnerable to CDK12 inhibition, which prompted us to put forward the hypothesis that blocking SEs through targeting CDK12 may diminish the relevant cellular traits (e.g., proliferation, survival and stemness), thereby eventually alleviating liver metastasis in CRC.

### 3.4 Blocking CDK12 attenuates liver metastasis in CRC

**FIGURE 4:**
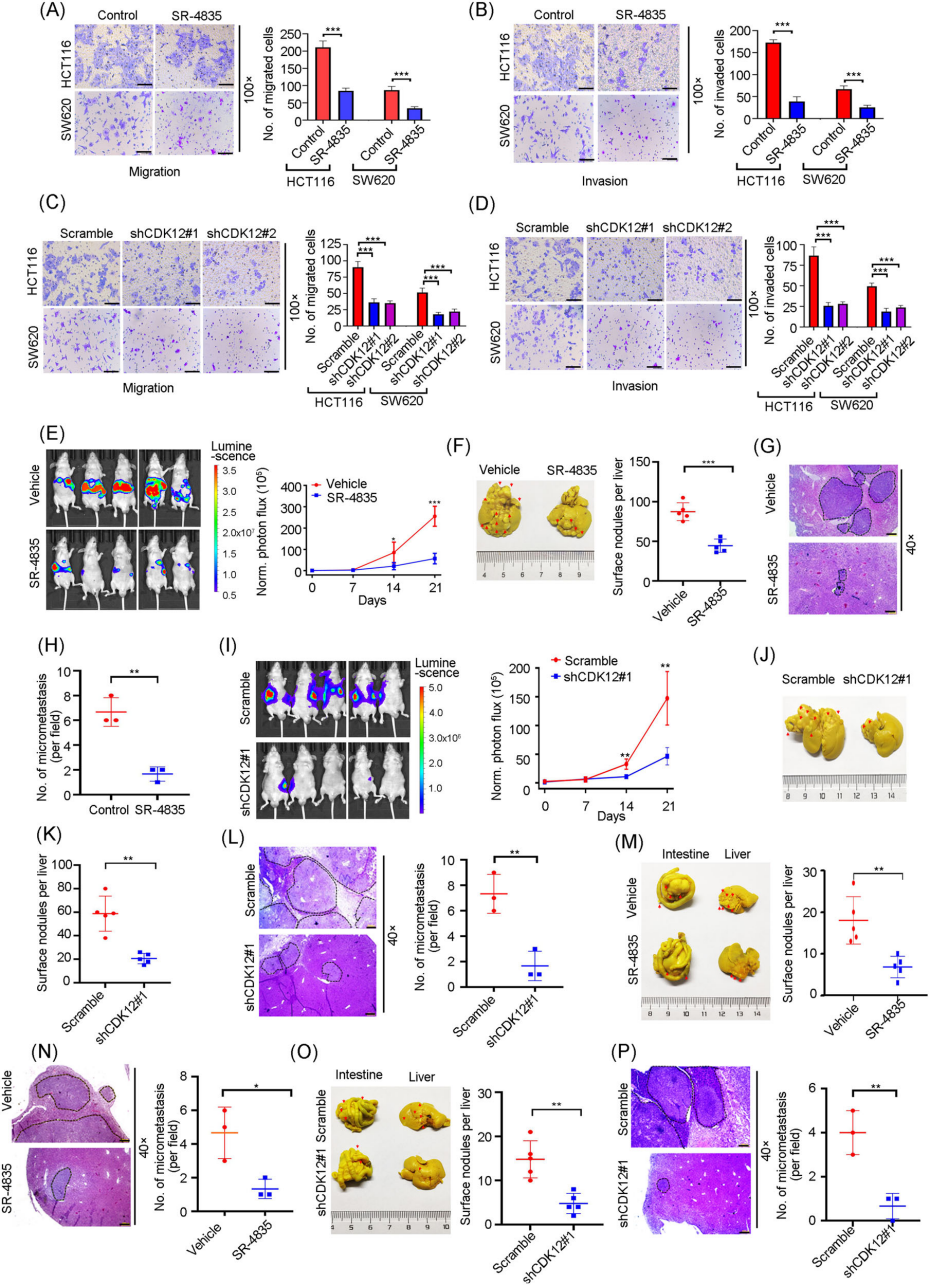
CDK12 inhibition suppresses hepatic metastasis in colorectal cancer (CRC). (A and B) After HCT116 or SW620 cells incubated with 100 nM SR‐4835 for 24 h, viable cells were counted and underwent transwell migration (A) or matrigel invasion (B) chamber assays. Representative images (left) for CRC cells and quantitative analysis (right) from three random fields were shown. Scale bar: 200 μm. (C and D) HCT116 or SW620 cells transfected with pLKO.1 (Scramble) or lentiviral shRNA against CDK12 (shCDK12) were subjected to transwell migration (C) or matrigel invasion (D) chamber assays. Representative images (left) for CRC cells and quantitative analysis (right) from three random fields were shown. Scale bar: 200 μm. (E) After intrasplenic injection of 5 × 10^6^ of HCT116‐luc cells for 24 h, the BALB/c nude mice were orally administrated with vehicle [10/90 dimethyl sulphoxide (DMSO)/30% hydroxypropyl‐b‐cyclodextrin in water] or 20 mg/kg SR‐4835 (5 days on, 2 days off per week) for 3 weeks. Left: representative images of mice by bioluminescence imaging (BLI) on day 21 were captured. Right: quantification of BLI photon flux every week post‐injection of HCT116‐luc cells. *n* = 5 per condition. (F) After mice treated with vehicle or SR‐4835 for 3 weeks, liver was isolated and fixed with Bouin's solution. Representative images of liver (left) and quantification of liver surface foci (right) were shown. Arrows indicated the distribution metastatic foci. *n* = 5 per group. (G and H) Representative images of haematoxylin–eosin (H&E) staining of liver section (G). Scale bar: 100 μm. Quantification of the number of liver metastatic foci in microscopic fields were shown (H). *n* = 3 per group. (I) HCT116‐luc cells stably transduced with Scramble or shCDK12 were intrasplenically injected in nude mice for 3 weeks. Liver metastasis was weekly analysed by BLI. Representative images on day 21 (left) and quantitative analysis (right) of photon flux were shown. *n* = 5 each group. (J and K) After mice received intrasplenic inoculation of 5 × 10^6^ of HCT116‐luc cells stably transduced with Scramble or shCDK12 for 3 weeks, livers were isolated and fixed with Bouin's solution. Representative images of liver (J) and quantification of liver surface foci (K) were shown. Arrows indicated the distribution metastatic foci. *n* = 5 per group. (L) Sections of liver were stained with H&E (left) and the number of liver micrometastases was counted with a dissection microscope (right). *n* = 3 each group. Scale bar: 100 μm. (M) Two weeks after 5 × 10^6^ HCT116 cells were subcutaneously injected into the flank of BALB/C nude mice, the tumours were excised, divided into small pieces with 2–3 mm in diameter and orthotopically transplanted into the nude mice. One day later, mice were randomly divided into two groups and administrated vehicle or SR‐4835 for 2 months. Left: representative gross photographs of the intestines and livers were shown. Arrows indicated the primary tumours in the intestines, and metastatic foci in the liver. Right: quantification of liver surface metastatic nodules was shown. *n* = 5 per condition. (N) Sections of the liver were stained with H&E (left) and the number of liver micrometastases was counted with a dissection microscope (right). *n* = 3 per condition. Scale bar: 100 μm. (O) After 5 × 10^6^ HCT116 cells stably transduced with Scramble or shCDK12 were subcutaneously injected into the flank of nude mice for 2 weeks, the tumours were excised, divided into small pieces with 2–3 mm in diameter and orthotopically transplanted into the nude mice for 2 months. Left: representative gross images of the intestines and livers were shown. Arrows indicated the primary tumours in the intestines, and metastatic foci in the liver. Right: quantification of liver surface metastatic nodules was presented. *n* = 5 each group. (P) Sections of the liver were stained with H&E (left) and the number of liver micrometastases was counted with a dissection microscope (right). *n* = 3 each group. Scale bar: 100 μm. ^*^ *p* < .05; ^**^ *p* < .01; ^***^ *p <* .001, Student's *t‐*test for results in (A), (B), (E), (F), (H), (I), (K)–(P). ^***^ *p <* .001, one‐way analysis of variance (ANOVA) with post hoc intergroup comparison by Tukey's test for results in (C) and (D)

To test the above hypothesis, we inhibited CDK12 by employing SR‐4835 or using specific shRNA, which can effectively block SE activity. Transwell analysis revealed that SR‐4835 significantly reduced the capacity of migration and invasion in HCT116 and SW620 cells (Figure 4A,B). Similarly, CDK12 knockdown in HCT116 and SW620 cells obviously diminished their migration and invasion abilities (Figures 4C,D and S2A). We next determined the important role of CDK12 in vivo with a liver metastasis model of CRC. BALB/c nude mice were orally administered SR‐4835 (20 mg/kg, 5 days on, 2 days off per week) for 3 weeks after intrasplenic inoculation with HCT116‐luc cells. BLI were weekly monitored with IVIS Spectrum system. The results showed that SR‐4835 administration significantly reduced the BLI signal intensity at liver zone on day 21 relative to vehicle‐treated mice (Figure 4E). As expect, the number of metastatic nodules on liver surface of mice upon SR‐4835 treatment were significantly decreased (Figure 4F). Accordingly, SR‐4835 treatment significantly decreased the number and size of metastatic foci in the liver, according to H&E staining analysis (Figure 4G,H). Consistent with SR‐4835 treatment, declined BLI signal intensity at liver zone on day 21 and the reduced number of metastatic nodules on liver were found in BALB/c nude mice intrasplenically injected CDK12‐silenced HCT116‐luc cells (Figures 4I–L and S2B).

Furthermore, an in vivo orthotopic implantation mouse model was employed to evaluate the function of CDK12 in liver metastasis of CRC. BALB/c nude mice orthotopically transplanted with the 2–3 mm small tumour pieces were orally administrated with SR‐4835 for approximately 2 months (20 mg/kg, 5 days on, 2 days off per week). The results showed that lessened distribution of tumour foci across the intestine was observed in SR‐4835‐treated mice compared with vehicle‐treated mice. More importantly, the metastatic nodules on liver surface of mice were evidently suppressed upon SR‐4835 administration (Figure 4M). Histologic examination also revealed that a lowered number and decreased size of metastatic nodules on the livers of mice were administrated with SR‐4835 (Figure 4N). Parallelly, attenuated distribution of tumour foci across the intestine and decreased metastatic foci in the liver were observed in nude mice orthotopically implanted with CDK12‐depleted HCT116 cells compared with mice bearing Scramble control cells (Figure 4O,P). These results support that blockade of SEs by CDK12 inhibition decreases liver metastasis in CRC.

### 3.5 Inhibition of CDK12 restricts cellular proliferation and outgrowth of xenografted cells in CRC

We then, respectively, explored which cellular features (e.g., proliferation, survival, CSCs) that were essential for tumour metastasise to distant organs were dampened by SR‐4835 treatment.

**FIGURE 5:**
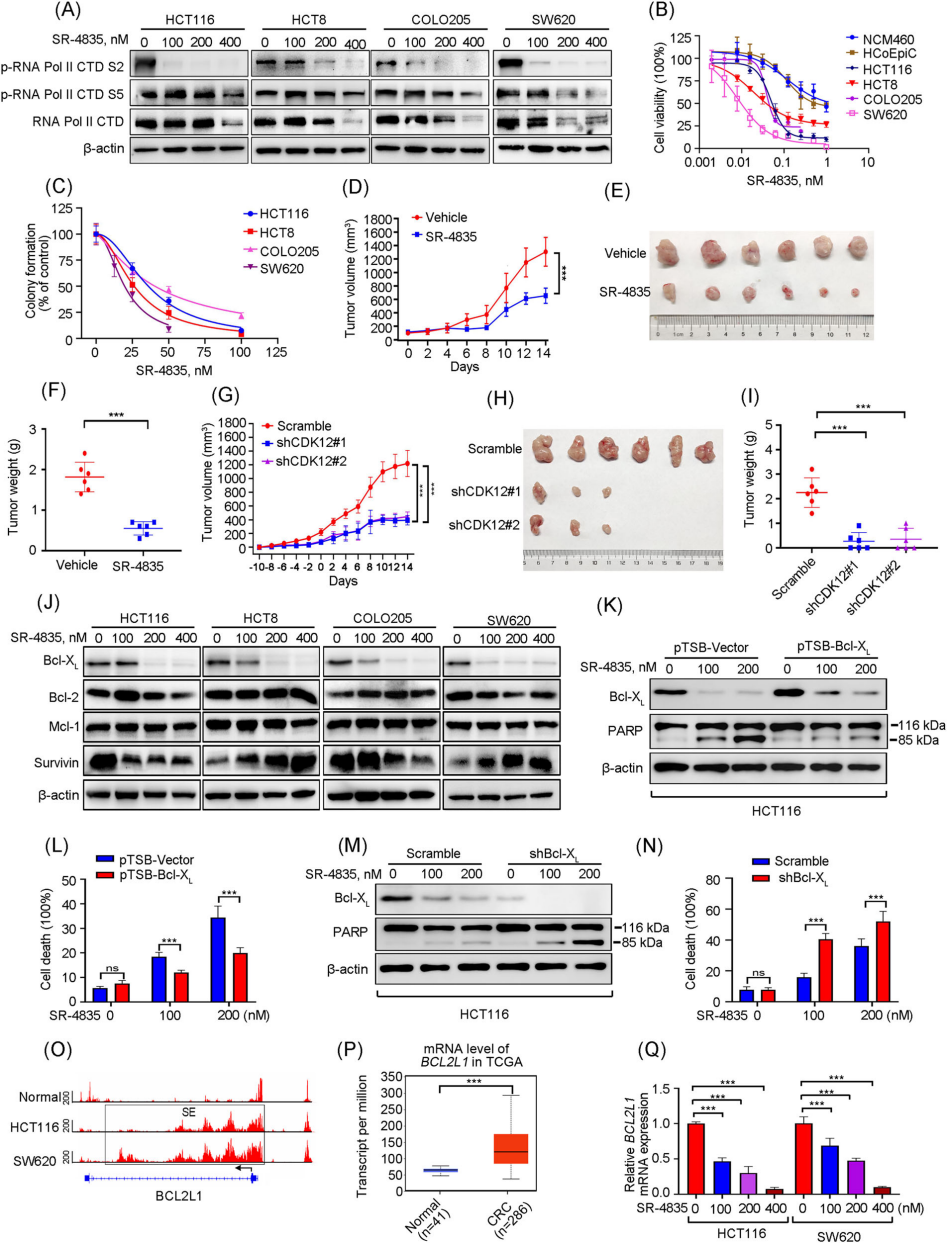
CDK12 inhibition restrains proliferation and survival of colorectal cancer (CRC) cells. (A) After CRC cells were treated with increasing concentrations of SR‐4835 for 24 h (HCT8 and COLO205 cells) or 48 h (HCT116 and SW620 cells), Western blotting analysis was performed. (B) Human normal colonic epithelial cells (HCoEpiC and NCM460) and CRC cells (HCT116, HCT8, COLO205 and SW620) were incubated with increasing doses of SR‐4835 for 68 h, and then cell viability was measured by CCK8 assay. (C) After pretreated with increasing concentrations of SR‐4835, CRC cells were cultured in soft agar to assess colony‐formation ability. (D–F) The effects of SR‐4835 on xenografted HCT116 cells were presented. Tumour size was plotted in the BALB/C nude mice treated with vehicle or 20 mg/kg SR‐4835 (orally 5 days on, 2 days off per week). *n* = 6 per group, 1 × 10^7^ cells per mouse (D). Representative images of tumours (E) and data of tumour weights from each group (F) were shown. (G–I) CDK12 knockdown inhibits outgrowth of xenografted HCT116 cells, *n* = 6 each group. Tumour growth curve was plotted in the nude mice bearing Scramble or CDK12‐silenced HCT116 cells (*n* = 6 per group, 1 × 10^7^ cells per mouse). Day 0 was denoted as the point that the tumour volume in the CDK12‐knockdown group reached ∼50 mm^3^, which was 10 days after cell inoculation (referred to as Day ‐10) (G). Representative images of tumours (H) and weights of tumours from each group (I) were shown. (J) The expression levels of apoptosis‐related proteins in SR‐4835‐treated CRC cells were detected by Western blotting assay. (K and L) After HCT116 cells were transfected with Bcl‐X_L_‐encoding constructs with or without SR‐4835 treatment, protein levels of PARP (K) and trypan blue exclusion assay were then detected (L). (M and N) After HCT116 cells were transfected with lentiviral shRNA against Bcl‐X_L_ with or without SR‐4835 treatment, the protein levels of PARP (M) and trypan blue exclusion assay (N) were then detected. (O) The signals of H3K27ac at *BCL2L1* (encoding Bcl‐X_L_) gene loci in normal colon tissue and CRC cells were plotted from the SEdb and dbCoRC database. (P) The mRNA levels of *BCL2L1* in CRC patients and normal adjacent colon tissues were obtained from The Cancer Genome Atlas Program (TCGA) in UALCAN website. (Q) Quantitative real‐time polymerase chain reaction (qRT‐PCR) experiment analysed the mRNA level of *BCL2L1* in SR‐4835‐treated HCT116 and SW620 cells. ns, not significant; ^***^ *p* < .001, Student's *t‐*test for results in (D), (F), (L), (N) and (P). ^***^ *p* < .001, one‐way analysis of variance (ANOVA) with post hoc intergroup comparison by Tukey's test for results in (G), (I) and (Q)

Western blotting analysis revealed that RNA Pol II CTD phosphorylation of S2 was obviously repressed by the CDK12 inhibitor SR‐4835 treatment for 24 or 48 h in CRC cells. Whereas, prolonged treatment (24 or 48 h) resulted in a decrease of initiation‐associated S5, possibly due to the reduced expression of total RNA Pol II (Figure 5A). We next used CCK8 assay to evaluate the effect of SR‐4835 on cellular proliferation. The results displayed that SR‐4835 effectively diminished cell viability of CRC cells with IC_50_ values of 15–68 nM (Figure 5B). Conversely, SR‐4835 showed 5–29‐fold less cytotoxicity in HCoEpiC and NCM460 cells with IC_50_ value of 320–430 nM (Figure 5B). Furthermore, SR‐4845 treatment remarkably inhibited the clonogenicity of CRC cells by using double layer soft agar system (Figure 5C). Meanwhile, depletion of CDK12 also induced significant retardation in cellular proliferation (Figure S3A,B) and in clonogenicity (Figure S3C) of CRC cells. These data indicate that inhibition of CDK12 effectively restrains growth of CRC cells with less cytotoxicity in vitro.

We further examined whether suppression of CDK12 abrogated the in vivo outgrowth of CRC cells. Therefore, xenograft mouse model bearing HCT116 cells was used. The results showed that the increased of tumour volume and tumour weight were significantly alleviated by SR‐4835 treatment (Figure 5D–F). In addition, the IHC against Ki67 showed that SR‐4835 treatment remarkably decreased proliferation (Figure S3D). We next obtained the cell lysates from the xenografted tumours, and to perform Western blotting analysis, the data showed that SR‐4835 administration resulted in a remarkable decrease in phosphorylation of RNA Pol II CTD at S2 instead of S5 (Figure S3E), indicative of the in vivo anti‐CDK12 power of SR‐4835. More importantly, the in vivo anti‐tumour activity of SR‐4835 was also observed in mice subcutaneously implanted with CDK12‐silenced HCT116 cells (Figures 5G–I and S3F). These results suggest that inhibition of CDK12 by SR‐4835 or abolished CDK12 expression with shRNA impedes cellular proliferation and outgrowth of xenografted cells in CRC.

### 3.6 BCL2L1 is a SE‐associated survival gene in CRC

The effect of SR‐4835 on apoptosis in CRC cells was performed. Annexin V‐PI dual staining based on flow cytometry analysis showed that SR‐4835 treatment induced dose‐dependent apoptosis in CRC cells (Figure S3G). As well, Western blotting results revealed that SR‐4835 trigged the activation of caspase‐3 and increased specific cleavage of PARP in CRC cells (Figure S3H). Of importantly, increased apoptosis was also detected by IHC staining with antibody against active caspase‐3 in SR‐4835‐administered HCT116 xenografts or CDK12‐silenced xenograft tumours (Figure S3D,F).

We next identified the expression of apoptosis‐related proteins to investigate the mechanism of apoptosis produced by SR‐4835 in CRC cells. The results showed a remarkable reduction in pro‐survival Bcl‐X_L_, while barely affecting the other apoptotic‐related proteins (e.g., Bcl‐2, Survivin, Mcl‐1) (Figure 5J). Of note, we found that SR‐4835 treatment suppressed the expression of Bcl‐X_L_ in tumour lysates from xenografts detected by Western blotting (Figure S3E). To further clarify the significance of Bcl‐X_L_ in the SR‐4835‐induced apoptosis, empty vector or human Bcl‐X_L_ plasmid‐expressed HCT116 cells were established and exposed to SR‐4835. We found that forced overexpression of Bcl‐X_L_ significantly impaired the SR‐4835‐induced apoptosis (Figure 5K,L), whereas knockdown of Bcl‐X_L_ increased the sensitivity of HCT116 cells to the SR‐4835‐induced apoptosis as evidenced by the protein change in cleavage of PARP and the percent of cell death determined by trypan blue exclusion (Figure 5M,N). These data suggest that CDK12 exerts pro‐survival role via regulation of Bcl‐X_L_.

To further investigate the mechanism that CDK12 modulates Bcl‐X_L_, we analysed the ChIP‐Seq signal of H3K27ac in normal colon tissue and CRC cells. The result revealed that the SE region at *BCL2L1* (encoding Bcl‐X_L_) gene loci in CRC cells was acquired relative to normal colon tissues (Figure 5O). Consistently, TCGA data showed that the *BCL2L1* mRNA levels were robustly increased in CRC tissues compared to healthy colon tissues, and positively correlated with short overall survival in CRC patients (Figures 5P and S4A). To confirm whether *BCL2L1* was regulated by SE, the CRISPR activation (CRISPRa) and CRISPR inhibition (CRISPRi) systems to recruit either a transcriptional activator or repressor complex to the individual SE peak with a catalytically dead Cas9 (dCas9) were employed.^21^, ^22^ The results revealed that use of the SAM complex to SE peaks of *BCL2L1* with four independent msgRNAs significantly increased the transcriptional and protein levels of Bcl‐X_L_ in CRC cells (Figure S4B–D). In contrast, the dCas9‐KRAB repressor complex to the same SE loci by sgRNAs significantly suppressed the expression of Bcl‐X_L_ (Figure S4B–D). We next explored how CDK12 regulated *BCL2L1*. SR‐4835 significantly inhibited the expression of *BCL2L1* revealed by qRT‐PCR analysis (Figure 5Q). Moreover, knockdown of CDK12 instead of CDK13, which shares highly conserved sequences with CDK12, remarkably reduced the mRNA and protein levels of Bcl‐X_L_ (Figure S4E,F), which indicates that the expression of Bcl‐X_L_ was specifically regulated by CDK12 but not CDK13. To further evaluate whether the expression of *BCL2L1* was regulated by CDK12 through SE, we performed ChIP‐qPCR assay and found that SR‐4835 treatment remarkably decreased the occupancy of CDK12 and RNA Pol II on the SE region of *BCL2L1* with no obvious change on the negative control region (NEG) (Figure S4G). Overall, these findings confirmed the regulatory role of SE in promoting *BCL2L1* expression. Finally, to explore the biological function of SE at *BCL2L1* gene loci, we performed experiments and found that the SAM system targeting individual SE peaks of *BCL2L1* significantly facilitated cellular proliferation, migration and invasion, while the dCas9‐KRAB repressor complex resulted in contrast results in CRC (Figure S4H,I). Collectively, these above results imply that SEs regulated by CDK12 play an important role in maintaining cellular malignant features that are essential for metastasis through promoting the high expression of *BCL2L1*.

### 3.7 Inhibition of CDK12 diminishes traits of CSCs

**FIGURE 6:**
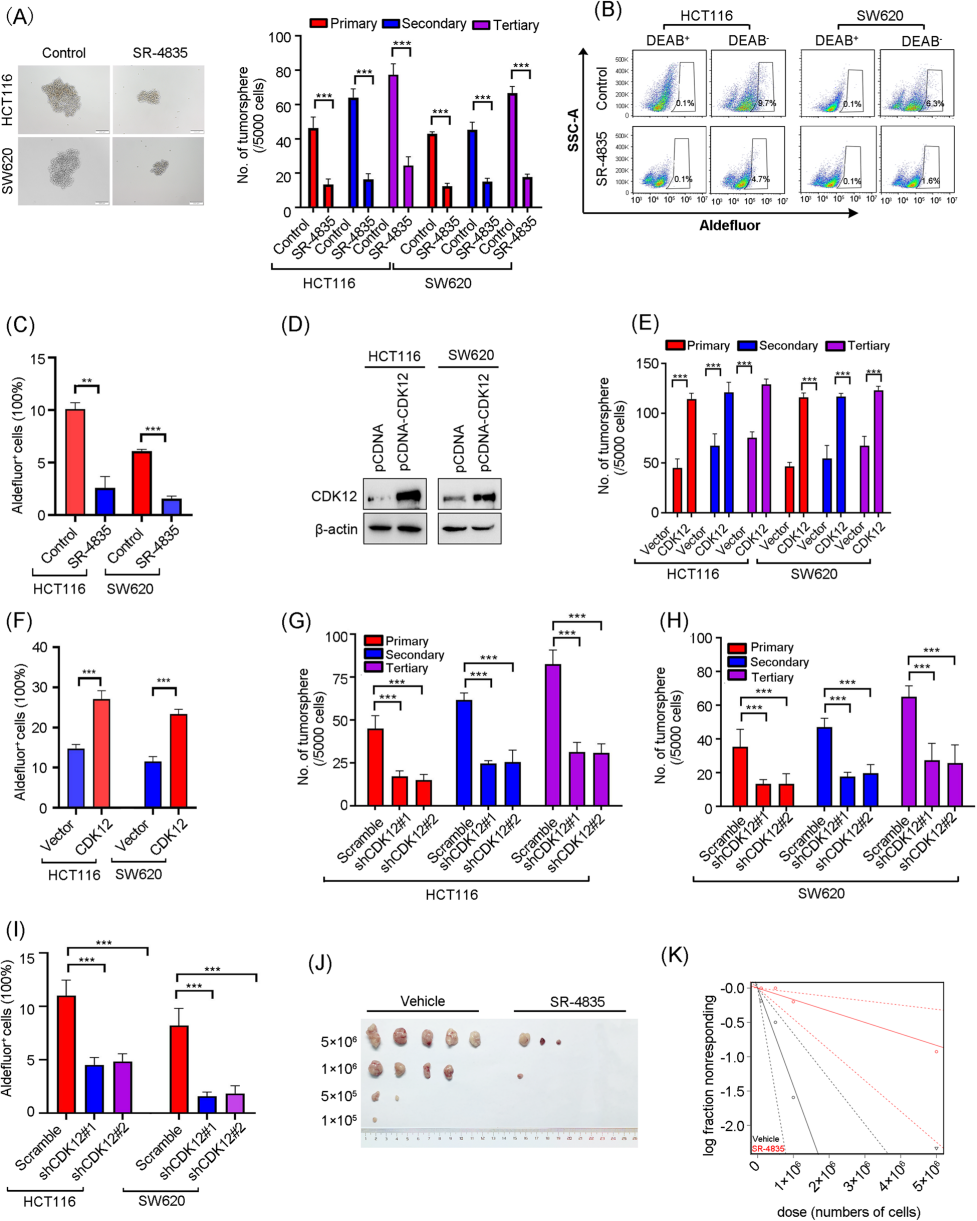
Targeting CDK12 impairs cancer stem‐like properties in colorectal cancer (CRC). (A) SR‐4835 repressed tumoursphere formation and serially replating ability in CRC cells. After HCT116 and SW620 cells treated with 100 nM SR‐4835 for 24 h, 5000 viable cells were resuspended in stem cell culture medium and plated in the ultralow‐attachment plates. On day 7, the number of tumoursphere were counted and photographed. Five thousand viable cells were then harvested and re‐plated per well for the secondary and tertiary rounds of tumoursphere formation, respectively. Representative images of tumoursphere (left) and quantification data (right) were shown. Scale bar: 100 μm. (B and C) SR‐4835 decreased the percentage of Aldefluor^+^ CRC cells. After HCT116 and SW620 cells were treated with 100 nM SR‐4835 for 24 h, the percentage of Aldefluor^+^ cells was determined by flow cytometry. Representative flow cytometry dot for Aldefluor^+^ CRC cells (B) and quantification data from three independent experiments (C) were shown. (D) The overexpression efficiency of CDK12 in HCT116 and SW620 cells was examined by Western blotting assay. (E) Forced overexpression of CDK12 enhanced tumoursphere formation and serially replating ability in CRC cells. (F) CDK12 overexpression boosted the percentage of Aldefluor^+^ CRC cells. (G and H) Silencing CDK12 attenuated tumoursphere formation and serially replating ability in HCT116 (G) and SW620 cells (H). (I) CDK12 knockdown decreased the percentage of Aldefluor^+^ CRC cells. (J and K) SR‐4835 treatment inhibited the frequency of CSCs in HCT116 cells examined by limiting dilution assay in nude mice, *n* = 5 per group. Representative image of tumours isolated from each group were shown (left) and the frequency of CSCs in CRC after SR‐4835 treatment was calculated (right). ^**^ *p* < .01; ^***^ *p* < .001, Student's *t‐*test for results in (A), (C), (E) and (F). ^***^ *p* < .001, one‐way analysis of variance (ANOVA) with post hoc intergroup comparison by Tukey's test for results in (G)–(I)

Given that CSCs undergo self‐renewal to facilitate tumour metastasis and recurrence, we wonder whether CDK12 inhibition affected traits of CSCs in CRC. Tumoursphere formation and serially replating assay were used to evaluate the self‐renewal capacity of CRC cells. The results revealed that exposure of SR‐4835 decreased the size and number of tumourspheres in serial passages (Figure 6A). Meanwhile, a reduced percentage of Aldefluor^+^ cell was exhibited in SR‐4835‐treated CRC cells, which was detected by flow cytometry analysis (Figure 6B,C).

In order to further ascertain the effect of CDK12 on CSCs properties, we forced overexpression of CDK12 in CRC cells (Figure 6D). Consistently, ectopic expression of CDK12 significantly boosted CSC properties as displayed the increased yields of tumoursphere (Figure 6E) and the elevated percentage of Aldefluor^+^ cells (Figure 6F). In contrast, silencing CDK12 significantly eliminated the ability of serial tumoursphere formation (Figure 6G,H) and repressed the proportion of Aldefluor^+^ cells (Figure 6I) in CRC cells. Moreover, the in vivo limiting dilution assay with HCT116 cells implanted into nude mice revealed that SR‐4835 treatment reduced CRC CSCs frequency 5.8‐fold (vehicle: 1.43 × 10^−6^; SR‐4835: 2.44 × 10^−7^) (Figure 6J,K and Table S5). These results suggest that blockade of SEs by inhibition of CDK12 eradicates the traits of CSCs in CRC.

### 3.8 *CCDC137* is a potential SE‐associated oncogene in CRC

**FIGURE 7:**
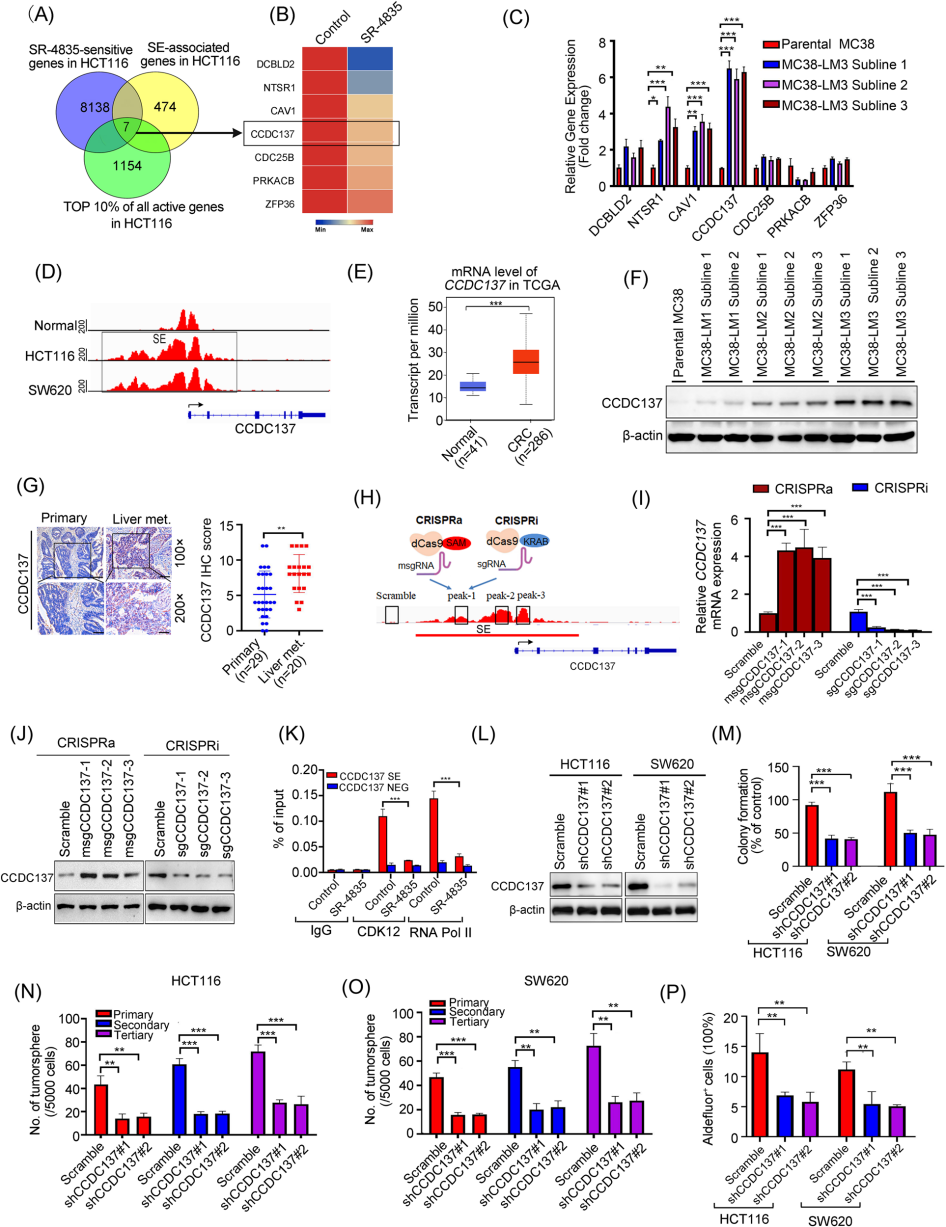
*CCDC137* is identified as a potential super‐enhancer (SE)‐associated oncogene in colorectal cancer (CRC) cells. (A) Venn diagram displayed overlap of SR‐4835‐sensitive transcripts, SE‐associated genes and the top 10% highest expressed genes in HCT116 cells. (B) Heatmap showed the RNA sequencing (RNA‐Seq) result of candidate SE‐associated genes in HCT116 cells after incubation with SR‐4835 for 6 h. (C) The mRNA levels of candidate SE‐associated genes were analysed by quantitative real‐time polymerase chain reaction (qRT‐PCR) in parental MC38 cells and the third round of highly liver metastatic MC38 (MC38‐LM3) subclones. (D) The signals of H3K27ac at *CCDC137* gene loci in normal colon tissue and CRC cells were plotted from the SEdb and dbCoRC database. (E) The transcriptional levels of *CCDC137* were shown in patients with CRC and normal adjacent tissues obtained from The Cancer Genome Atlas Program (TCGA) database. (F) The protein levels of CCDC137 were analysed by Western blotting assay in parental MC38 cells, the first, second and third round of highly liver metastatic MC38 (MC38‐LM1, MC38‐LM2 and MC38‐LM3) subclones. (G) Representative immunohistochemistry (IHC) images of CCDC137 expression in primary CRC tissues and liver metastasis CRC tissues (left) and IHC score determined by multiply area score and light score (right) were shown. Scale bar: 200 μm (100×), 100 μm (200×). (H) The schematic shows targeting SE region of *CCDC137* with three individual gRNAs (gCCDC137‐1 to gCCDC137‐3) utilising a CRISPR activation (CRISPRa) system with the transcriptional activator (SAM)‐associated catalytically dead Cas9 (dCas9) or a CRISPR inhibition (CRISPRi) system with the repressor complex (KRAB‐MeCP2)‐associated dCas9. (I) The qRT‐PCR results showed the mRNA levels of *CCDC137* were elevated or reduced by employing the CRISPRa or CRISPRi system targeting individual SE peaks in HCT116 cells. (J) Western blotting showed the protein levels of CCDC137 were upregulated or downregulated by introducing the CRISPRa or CRISPRi system targeting individual SE peaks in HCT116 cells. (K) ChIP‐qPCR results showed that treatment of SR‐4835 decreased the occupancy of CDK12 and RNA polymerase II (Pol II) on the SE region of *CCDC137* with no obvious alteration on the negative control region (NEG) in HCT116 cells. (L) The knockdown efficiency of CCDC137 in HCT116 and SW620 cells were examined by Western blotting. (M) Colony formation was detected in CDK12‐depleted HCT116 and SW620 cells. (N and O) Silencing CCDC137 attenuated tumoursphere formation and serially replating ability in HCT116 (N) and SW620 cells (O). (P) CCDC137 knockdown decreased the percentage of Aldefluor^+^ cells in HCT116 and SW620 cells. ^**^ *p* < .01; ^***^ *p* < .001, one‐way analysis of variance (ANOVA) with post hoc intergroup comparison by Tukey's test for results in (C), (I), (M)–(P). ^**^ *p <* .01; ^***^ *p* < .001, Student's *t‐*test for results in (E), (G) and (K)

To identify candidate SE‐associated oncogenes contribute to liver metastasis in CRC, we searched for candidate genes based on: (i) they are associated with SEs in CRC cells; (ii) they are ranked the top 10% of actively expressed genes in RNA‐Seq results; (iii) their transcriptional level is selectively sensitive to low dose of SR‐4835 treatment (decreased more than 1.5‐fold change) (Figure 7A). As a result, seven candidate genes were obtained, namely, *DCBLD2*, *NTSR1*, *CAV1*, *CCDC137*, *CDC25B*, *PRKACB* and *ZFP36* (Figure 7B). The qRT‐PCR assay confirmed that these seven genes were significantly decreased in both HCT116 and SW620 cells after CDK12 inhibition by SR‐4835 treatment or silencing CDK12 with lentiviral shRNA (Figure S5A,B). Of note, the expression levels of *CCDC137* out of the seven genes was most abundant as examined by qRT‐PCR analysis in the MC38‐LM3 sublines relative to the parental MC38 cells (Figure 7C). In addition, among the seven genes, *DCBLD2*,^35^ *NTSR1*,^29^ *CAV1*,^36^ *CDC25B*,^37^ *PRKACB* ^38^ and *ZFP36* ^39^ have been demonstrated to be involved in the malignant phenotype of CRC, while the function of CCDC137 in CRC remains unknown. Thus, we mainly focused on analysis of CCDC137 in the following study.

To confirm whether *CCDC137* is a candidate SE‐associated oncogene, the ChIP‐Seq signal of H3K27ac in normal colon tissue and CRC cells were analysed in the SE databases. The result showed that the SE region of *CCDC137* in CRC cells was acquired relative to normal colon tissues (Figure 7D). Accordantly, the higher level of CCDC137 was found in CRC tissues relative to normal tissues in TCGA database (Figure 7E). Moreover, the abundant levels of CCDC137 were found in MC38‐LM3 sublines with highly hepatic metastatic ability (Figure 7F) and in liver metastatic CRC patients as revealed by IHC assay (Figure 7G) and Western blotting (Figure S5C). We further adopted the CRISPRa and CRISPRi systems to target the individual SE peaks of *CCDC137* to evaluate the transcriptional regulatory role of SE. The data revealed that the CRISPRa system significantly activated the transcriptional and protein levels of CCDC137, while the CRISPRi systems exerted the opposite effect (Figure 7H–J). We next verify whether the expression of CCDC137 was regulated by CDK12. The data from qRT‐PCR and Western blotting revealed that CDK12 suppression with SR‐4835 or shRNA significantly inhibited the in vitro and in vivo expression of CCDC137 (Figure S5A,B,D–F). Moreover, CCDC137 knockdown nearly had no effect on the expression of CDK12, which suggests CDK12 is the regulator of CCDC137 (Figure S5G). Furthermore, disruption of CDK13 had little effect on the mRNA and protein levels of CCDC137 (Figure S5H,I), which indicates that the expression of CCDC137 was specifically regulated by CDK12 but not CDK13. To further evaluate the regulatory role of CDK12 in SE loci, we performed ChIP‐qPCR assay and found that SR‐4835 treatment remarkably decreased the occupancy of CDK12 and RNA Pol II on the SE region of *CCDC137* with no obvious change on the NEG (Figure 7K). Together, these data suggest that CCDC137 is a SE‐associated gene modulated by CDK12 in CRC.

To figure out the biological function of CCDC137, TCGA database was analysed. It was shown that the mRNA levels of CCDC137 were not only increased with advanced CRC stages (Figure S5J), but also positively associated with lymph node metastatic stages of CRC (Figure S5K). Moreover, the overexpressed CCDC137 was positively correlated with short disease‐free survival in patients with CRC (Figure S5L). We further generated CCDC137‐disrupted CRC cell with CRISPRi system or lentiviral shRNA (Figure 7J,L). The results showed that CCDC137 suppression greatly decreased proliferation of CRC cells (Figure S5M–O) and colony formation assay (Figure 7M), respectively. Besides, inhibiting CCDC137 significantly obviated CSC properties as revealed by repressed serial tumoursphere formation (Figure 7N,O) and the percentage of Aldefluor^+^ cells (Figure 7P). Collectively, these findings indicate that CCDC137 may be a functionally critical SE‐associated oncogene in CRC.

### 3.9 CDK12 regulates CCDC137 to facilitate the characteristics of CSCs and liver metastasis in CRC

**FIGURE 8:**
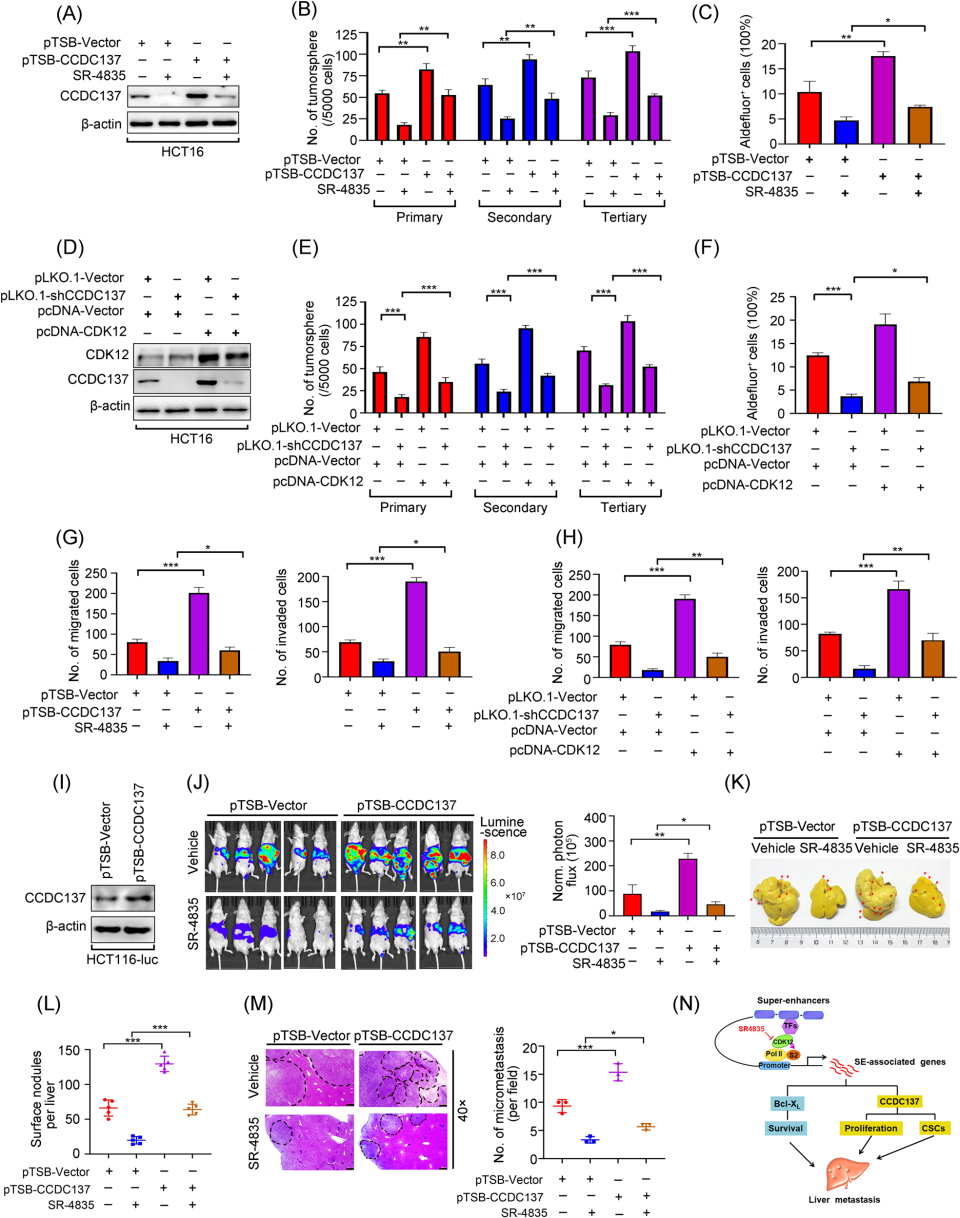
CCDC137 reverses the downregulated cancer stem cells (CSCs) traits and liver metastasis mediated by CDK12 inhibition in colorectal cancer (CRC) cells. (A–C) HCT116 cells stably expressing of CCDC137 cDNA (pTSB‐CCDC137) and empty vector (pTSB‐Vector) were treated with 100 nM SR‐4835 for 24 h, followed by Western blotting analysis (A), tumoursphere formation assay (B) and Aldefluor assay (C), respectively. (D) The efficiency of CCDC137 silence in CDK12‐overexpressed HCT116 cells were verified by Western blotting assay. (E) Knockdown of CCDC137 rescued the boosted capacity of tumoursphere formation mediated by CDK12 overexpressing in HCT116 cells. (F) Silence of CCDC137 reduced the increased percentage of Aldefluor^+^ cells mediated by CDK12 overexpressing in HCT116 cells. (G) HCT116 cells stably expressing of pTSB‐CCDC137 and pTSB‐Vector were treated with 100 nM SR‐4835 for 24 h, and then subjected to migration (left) and invasion (right) assay. (H) Depletion of CCDC137 rescued the migration (left) and invasion (right) ability in CDK12‐overexpressing HCT116 cells. (I) The overexpression efficiency of CCDC137 in HCT116‐luc cells were examined by Western blotting assay. (J) Nude mice were intrasplenically injected with 5 × 10^6^ HCT116‐luc cells stably expressing pTSB‐CCDC137 or pTSB‐Vector, followed by orally administrated with vehicle or 20 mg/kg SR‐4835 (5 days on, 2 days off per week) for 3 weeks. Representative images of photon flux (left) and quantification of photon flux of liver metastases in nude mice on day 21 (right) were shown. *n* = 5 each group. (K and L) After mice treated with vehicle or SR‐4835 for 3 weeks, livers were isolated and fixed with Bouin's solution. Representative images of liver (K) and quantification of liver surface foci (L) were shown. Arrows indicated the distribution metastatic foci. *n* = 5 per group. (M) Representative images of haematoxylin–eosin (H&E) staining of liver section (left) and quantification of the number of liver metastatic foci in microscopic fields (right) were displayed. *n* = 3 each group. Scale bar: 100 μm. (N) Proposed working model of targeting CDK12 effect on liver metastasis of CRC. ^*^ *p* < .05; ^**^ *p* < .01; ^***^ *p* < .001, one‐way analysis of variance (ANOVA) with post hoc intergroup comparison by Tukey's test for results in (B), (C), (E)–(H), (L) and (M)

Given that CCDC137 knockdown eliminated CSCs properties, we tested whether CCDC137 was implicated in the CDK12‐mediated maintenance of CSCs. HCT116 cells transducing with lentiviral CCDC137‐encoding construct were treated with SR‐4835, followed by Western blotting detection and CSCs traits evaluation. The results suggested that forced expression of CCDC137 boosted the capacity of tumoursphere formation and the percentage of Aldefluor^+^ cells compared with vector‐expressed cells (Figure 8A–C). Moreover, ectopic expression of CCDC137 abrogated the SR‐4835‐induced reduction in serial tumoursphere formation capacity and Aldefluor^+^ cells proportion (Figure 8A–C). Conversely, CCDC137 silencing attenuated the overexpression of CDK12‐induced elevation of the serial tumoursphere formation capacity and the Aldefluor^+^ cells percentage (Figure 8D–F).

We further conducted experiments to determine the effect of CCDC137 on metastasis in CRC. Migration and invasion assays indicated that forced overexpression of CCDC137 with pTSB‐CCDC137 cDNA or CRISPRa system enhanced the capabilities of migration and invasion in HCT116 cells compared with vector‐expressed cells (Figures 8G and S5P). Besides, ectopic overexpression of CCDC137 rescued the abrogated capabilities of migration and invasion regulated by SR‐4835 in HCT116 cells (Figure 8G). In contrast, inhibition of CCDC137 by lentiviral shRNA or CRISPRi system effectively decreased the migratory and invasive capabilities in HCT116 cells (Figures 8H and S5P). Moreover, CCDC137 silencing reversed CDK12 overexpression‐mediated increased capabilities of migration and invasion (Figure 8H). Furthermore, the in vivo hepatic metastatic model by intrasplenic implantation of HCT116‐luc cells in nude mice was conducted. The results unveiled that stably transduced with CCDC137‐encoding construct led to a significant elevation in the BLI intensity and in the number and size of liver surface nodules (Figure 8I–M). Meanwhile, CCDC137 overexpression also displayed a strong effect to reverse the SR‐4835‐mediated inhibition of these two parameters of liver metastasis (Figure 8I–M). In summary, these results suggest that CCDC137 is essential for CDK12 to maintain CSCs traits and facilitate liver metastasis in CRC.

### 3.10 CDK12 expression is positively correlated with CCDC137 in CRC patients

We further determined the correlation between CDK12 and CCDC137 in human CRC tissues. Analysis of TCGA database showed that a significant correlation between CDK12 and CCDC137 was existed in CRC patients (Figure S6A). Moreover, the mRNA levels of CDK12 were significantly positively associated with CCDC137 mRNA levels in the 18 CRC biopsies as reflected by qRT‐PCR assay (Figure S6B,C). Notably, Western blotting detection indicated that both the expression of CDK12 and CCDC137 were evidently upregulated compared with the paired adjacent normal tissues, and the protein levels of CDK12 were positively correlated with CCDC137 expression (Figure S6D). In addition, IHC staining of the CRC tissues further verified that CDK12 expression was positively correlated with CCDC137 expression (Figure S6E). The observations imply that CDK12 regulation of CCDC137 may be crucial to the development of CRC.

## 4 DISCUSSION

Clarification of mechanism underlie liver metastasis and identification novel candidate targets for CRC treatment have long been desired. In this study, we found that SEs were invovled in the malignant phenotype maintenance and related with liver metastasis in CRC. CDK12 as an required role of the SE machinery was upregulated in highly liver metastatic CRC cells and CRC patients. Perturbation of the SE‐associated transcriptional addiction by CDK12 inhibition effectively eradicated metastatic‐associated cellular features, such as proliferation, survival and CSCs in vitro and in vivo. Furthermore, we identified that *BCL2L1* and *CCDC137* were critical SE‐associated oncogenes by strengthening cellular survival, proliferation and CSCs traits in CRC.

CDK12‐modulated phosphorylation of S2 on the CTD of RNA Pol II facilitates transcriptional elongation. In line with this, our results indicated that after inhibition of CDK12 with the inhibitor, SR‐4835 or knockdown with lentivirus shRNA, the amout of phosphorylation of S2 on the CTD of RNA Pol II was remarkably decreased. In particular, SE‐associated genes were striking downregulated upon SR‐4835 treatment or CDK12 depletion, which may confer the sensitivity to CDK12 inhibition in CRC. Consistent with our observations, Zhang et al.^14^ reported that inactivation of CDK12 by small molecule THZ531 substantially abrogates SE‐associated transcriptional addiction in T‐cell acute lymphoblastic leukaemia. Moreover, CDK12 intervention affects the DNA damage response genes expression in various cancer types including TNBC,^15^ hepatocellular carcinoma^17^ and osteosarcoma,^1^ resulting in increased DNA damage. Consistently, SE‐associated genes and SR‐4835‐sensitive genes were found to be enriched in DNA damage as displayed by GO analysis in CRC cells. In addition, a strong DNA damage increase was also detected in SR‐4835‐treated CRC cells and HCT116 subcutaneous tumours from either SR‐4835‐treatment or the CDK12‐knockdown group as reflected by the protein levels of γ‐H2AX (Figure S7A–C). There are numerous studies regarding the synergy between silencing CDK12 and PARP inhibitors or other DNA‐damaging agents, including olaparib, irinotecan in breast cancer.^15^, ^16^ Furthermore, in combination with the immune checkpoint inhibitors such as anti‐PD1, SR‐4835 markedly enhances the killing effect of tumour cells through enhancing the activation and function of dendritic cells and T cells.^40^ In agreement with these results, we found a strong synergistic activity against CRC between SR‐4835 and olaparib or PD‐L1 inhibitor BMS‐202 (Figure S8A–D). Thus, inhibition of CDK12 by SR‐4835 in combination with either PARP inhibitor or immune checkpoint inhibitor may be a promising strategy for CRC patients.

An important highlight of this study is that SEs drive liver metastasis in CRC. Since SEs were initially found to be implicated in liver metastasis through integrative analysis of SE database and GEO database, we then determined to explore the functional role of SEs in CRC liver metastasis. Blocking SEs by CDK12 inhibition significantly diminished liver metastasis by employing the liver metastatic mouse model and orthotopic mouse metastatic model, which confirmed SEs contributing to liver metastasis in CRC. Mechanically, SEs enhanced the transcription of the SEs‐associated genes of *CCDC137* and *BCL2L1* to boost cellular proliferation, CSCs and survival in CRC cells, respectively. In accordance with our findings, Yokoyama et al.^41^ reported that inhibiting SEs by BRD4 inhibitors JQ1 abrogates expression levels of ALDH1A1 and other stem‐related genes, followed by decreasing chemotherapy resistance and tumour relapse in ovarian cancer. Zhang et al.^42^ documented that blocking SEs by CDK7 inhibitor THZ2 suppresses the growth and metastasis in osteosarcoma. Ye's research group^43^ and Dong et al.^44^ also demonstrated that disrupting SEs with JQ1 effectively eliminates CSCs and halts metastasis in squamous cell carcinoma.

A crucial characteristic of cancer is a strengthened capacity for survival, which also is necessary for development of metastasis.^25^ Dysregulation of the Bcl‐2 family members is the main mechanism of pro‐survival or resistance to apoptosis. Numerous cancers have increased levels of Bcl‐X_L_, a member of the Bcl‐2 family, which has a positive correlation with tumour grade and metastasis.^27^, ^45^ It was found that Bcl‐X_L_ instead of Bcl‐2 or Mcl‐1 is required for cell survival and outgrowth during the adenoma‐to‐carcinoma sequence of CRC.^27^ Jin et al.^45^ reported that Bcl‐X_L_ inhibition resulted in a decrease in cellular survival, eventually repressing forming overt metastases in uveal melanoma. In accord with previous studies, in samples of CRC relative to normal colon tissues, Bcl‐X_L_ was overexpressed, and this was positively linked with a worse prognosis in CRC patients. Moreover, we found that Bcl‐X_L_ was driven by SE and significantly reduced at transcriptional level after CDK12 inhibition with SR‐4835. Knockdown Bcl‐X_L_ by shRNA increased sensitivity of CRC cells to SR‐4835, whereas forced overexpression of Bcl‐X_L_ attenuated apoptosis induced by SR‐4835. Hence, our results suggest that Bcl‐X_L_ may offer an attractive therapeutic target in metastatic CRC.

We further identified that CCDC137 driven by SE serves as a critical oncogene to promote liver metastasis in CRC. CCDC137, as one vital member of coiled‐coil domain containing (CCDC) family proteins, was overexpressed and positively associated with worse prognosis in various tumour types, such as liver hepatocellular carcinoma, lower grade glioma and prostate adenocarcinoma.^46^ Similarly, we observed that CCDC137 expression was elevated in liver metastatic CRC patients and positively correlated with advanced tumour stages and lymph node metastatic stages of CRC. Furthermore, the overexpressed CCDC137 was significantly correlated with short disease‐free survival as shown in TCGA database. Importantly, previous studies demonstrated that CCDC family proteins can promote proliferation, CSCs and metastasis of cancer cells.^47^, ^48^ For example, CCDC80 is the core regulator for self‐renew in haematopoietic stem/progenitor cell,^47^ which functions as a stemness biomarker in CRC.^48^ In addition, Yang et al.^49^ have reported that CCDC25 enhances metastasis via activation of the ILK‐β‐parvin pathway in tumour cells. Therefore, it is conceivable that CCDC137 might promote proliferation, CSC characteristics and metastasis in CRC. As expect, we found that CCDC137 promoted liver metastasis by elevating proliferation and traits of CSCs in CRC cells. Abolishing CCDC137 elicited an effective elimination of CSCs, providing a therapeutic opportunity in CRC treatment.

The current study has certain limitations. For instance, the underlying mechanism of upregulated expression of CDK12 in CRC is still not clear. Only ∼8% cases of CRC in TCGA database showed aberrant CDK12 genomic alteration, including gene mutations, amplifications with an unknown effect on its expression.^50^ Other potential mechanisms (e.g., microRNA regulation, protein stability alteration) for CDK12 dysregulation should to be further investigated. Additionally, we found that CCDC137 was an SE‐associated oncogene and knockdown of CCDC137 inhibited proliferation and self‐renewal capacity in CRC cells. However, further research is required to determine the precise molecular mechanism by which CCDC137 regulates cellular proliferation and stemness.

In conclusion, we discovered that SE landscape was implicated in liver metastasis of CRC. CDK12 as the critical SE complex component was overexpressed in liver metastatic CRC patients and associated with poor prognosis. Suppression of CDK12 significantly impaired liver metastasis through blocking the transcription of SE‐associated genes in CRC. Furthermore, *BCL2L1* and *CCDC137* were found to be the key downstream SE‐associated genes of CDK12 to regulate metastasis‐related cellular features such as proliferation, survival and CSCs (Figure 8N). Targeting CDK12 and SE‐associated oncogenic transcripts may be a potential therapeutic strategy to inhibit hepatic metastasis in CRC.

## CONFLICT OF INTEREST

The authors declare they have no conflicts of interest.

## Supporting information

Supporting Information

## References

[1] Bayles I , Krajewska M , Pontius WD , et al. Ex vivo screen identifies CDK12 as a metastatic vulnerability in osteosarcoma. J Clin Invest. 2019;129(10):4377‐4392. 31498151 10.1172/JCI127718 PMC6763270

[2] Valastyan S , Weinberg RA . Tumor metastasis: molecular insights and evolving paradigms. Cell. 2011;147(2):275‐292. 22000009 10.1016/j.cell.2011.09.024 PMC3261217

[3] Chaffer CL , Weinberg RA . A perspective on cancer cell metastasis. Science. 2011;331(6024):1559‐1564. 21436443 10.1126/science.1203543

[4] Massagué J , Obenauf AC . Metastatic colonization by circulating tumour cells. Nature. 2016;529(7586):298‐306. 26791720 10.1038/nature17038 PMC5029466

[5] Roe JS , Hwang CI , Somerville TDD , et al. Enhancer reprogramming promotes pancreatic cancer metastasis. Cell. 2017;170(5):875‐888.e820. 28757253 10.1016/j.cell.2017.07.007 PMC5726277

[6] Pott S , Lieb JD . What are super‐enhancers? Nat Genet. 2015;47(1):8‐12. 25547603 10.1038/ng.3167

[7] Zhou J , Wang S , Nie D , et al. Super‐enhancer landscape reveals leukemia stem cell reliance on X‐box binding protein 1 as a therapeutic vulnerability. Sci Transl Med. 2021;13(612):eabh3462. 34550724 10.1126/scitranslmed.abh3462

[8] Jiang YY , Lin DC , Mayakonda A , et al. Targeting super‐enhancer‐associated oncogenes in oesophageal squamous cell carcinoma. Gut. 2017;66(8):1358‐1368. 27196599 10.1136/gutjnl-2016-311818 PMC5912916

[9] Chipumuro E , Marco E , Christensen CL , et al. CDK7 inhibition suppresses super‐enhancer‐linked oncogenic transcription in MYCN‐driven cancer. Cell. 2014;159(5):1126‐1139. 25416950 10.1016/j.cell.2014.10.024 PMC4243043

[10] Eliades P , Abraham BJ , Ji Z , et al. High MITF expression is associated with super‐enhancers and suppressed by CDK7 inhibition in melanoma. J Invest Dermatol. 2018;138(7):1582‐1590. 29408204 10.1016/j.jid.2017.09.056 PMC6019629

[11] Tsang FH , Law CT , Tang TC , et al. Aberrant super‐enhancer landscape in human hepatocellular carcinoma. Hepatology. 2019;69(6):2502‐2517. 30723918 10.1002/hep.30544

[12] Christensen CL , Kwiatkowski N , Abraham BJ , et al. Targeting transcriptional addictions in small cell lung cancer with a covalent CDK7 inhibitor. Cancer Cell. 2014;26(6):909‐922. 25490451 10.1016/j.ccell.2014.10.019 PMC4261156

[13] Krajewska M , Dries R , Grassetti AV , et al. CDK12 loss in cancer cells affects DNA damage response genes through premature cleavage and polyadenylation. Nat Commun. 2019;10(1):1757. 30988284 10.1038/s41467-019-09703-y PMC6465371

[14] Zhang T , Kwiatkowski N , Olson CM , et al. Covalent targeting of remote cysteine residues to develop CDK12 and CDK13 inhibitors. Nat Chem Biol. 2016;12(10):876‐884. 27571479 10.1038/nchembio.2166 PMC5033074

[15] Quereda V , Bayle S , Vena F , et al. Therapeutic targeting of CDK12/CDK13 in triple‐negative breast cancer. Cancer Cell. 2019;36(5):545‐558.e547. 31668947 10.1016/j.ccell.2019.09.004

[16] Iniguez AB , Stolte B , Wang EJ , et al. EWS/FLI confers tumor cell synthetic lethality to CDK12 inhibition in ewing sarcoma. Cancer Cell. 2018;33(2):202‐216.e206. 29358035 10.1016/j.ccell.2017.12.009 PMC5846483

[17] Wang C , Wang H , Lieftink C , et al. CDK12 inhibition mediates DNA damage and is synergistic with sorafenib treatment in hepatocellular carcinoma. Gut. 2020;69(4):727‐736. 31519701 10.1136/gutjnl-2019-318506

[18] Dieter SM , Siegl C , Codó PL , et al. Degradation of CCNK/CDK12 is a druggable vulnerability of colorectal cancer. Cell Rep. 2021;36(3):109394. 34289372 10.1016/j.celrep.2021.109394

[19] Liu S , He L , Wu J , et al. DHX9 contributes to the malignant phenotypes of colorectal cancer via activating NF‐κB signaling pathway. Cell Mol Life Sci. 2021;78(24):8261‐8281. 34773477 10.1007/s00018-021-04013-3 PMC11072136

[20] Wei F , Zhang T , Deng SC , et al. PD‐L1 promotes colorectal cancer stem cell expansion by activating HMGA1‐dependent signaling pathways. Cancer Lett. 2019;450:1‐13. 30776481 10.1016/j.canlet.2019.02.022

[21] Konermann S , Brigham MD , Trevino AE , et al. Genome‐scale transcriptional activation by an engineered CRISPR‐Cas9 complex. Nature. 2015;517(7536):583‐588. 25494202 10.1038/nature14136 PMC4420636

[22] Thakore PI , D'Ippolito AM , Song L , et al. Highly specific epigenome editing by CRISPR‐Cas9 repressors for silencing of distal regulatory elements. Nat Methods. 2015;12(12):1143‐1149. 26501517 10.1038/nmeth.3630 PMC4666778

[23] Jiang Y , Qian F , Bai X , et al. SEdb: a comprehensive human super‐enhancer database. Nucleic Acids Res. 2019;47(D1):D235‐D243. 30371817 10.1093/nar/gky1025 PMC6323980

[24] Huang M , Chen Y , Yang M , et al. dbCoRC: a database of core transcriptional regulatory circuitries modeled by H3K27ac ChIP‐seq signals. Nucleic Acids Res. 2018;46(D1):D71‐D77. 28977473 10.1093/nar/gkx796 PMC5753200

[25] Dai W , Liu S , Wang S , et al. Activation of transmembrane receptor tyrosine kinase DDR1‐STAT3 cascade by extracellular matrix remodeling promotes liver metastatic colonization in uveal melanoma. Signal Transduct Target Ther. 2021;6(1):176. 33976105 10.1038/s41392-021-00563-x PMC8113510

[26] Tseng W , Leong X , Engleman E . Orthotopic mouse model of colorectal cancer. J Vis Exp. 2007;10:484. 10.3791/484 PMC2557075 18989400

[27] Ramesh P , Lannagan TRM , Jackstadt R , et al. BCL‐XL is crucial for progression through the adenoma‐to‐carcinoma sequence of colorectal cancer. Cell Death Differ. 2021; 28(12): 3282‐3296. 34117376 10.1038/s41418-021-00816-w PMC8630104

[28] Lu B , He Y , He J , et al. Epigenetic profiling identifies LIF as a super‐enhancer‐controlled regulator of stem cell‐like properties in osteosarcoma. Mol Cancer Res. 2020; 18(1): 57‐67. 31615908 10.1158/1541-7786.MCR-19-0470

[29] Christou N , Blondy S , David V , et al. Neurotensin pathway in digestive cancers and clinical applications: an overview. Cell Death Dis. 2020; 11(12): 1027. 33268796 10.1038/s41419-020-03245-8 PMC7710720

[30] Fleming NI , Jorissen RN , Mouradov D , et al. SMAD2, SMAD3 and SMAD4 mutations in colorectal cancer. Cancer Res. 2013; 73(2): 725‐735. 23139211 10.1158/0008-5472.CAN-12-2706

[31] Soutourina J . Transcription regulation by the Mediator complex. Nat Rev Mol Cell Biol. 2018; 19(4): 262‐274. 29209056 10.1038/nrm.2017.115

[32] Stathis A , Bertoni F . BET proteins as targets for anticancer treatment. Cancer Discov. 2018; 8(1): 24‐36. 29263030 10.1158/2159-8290.CD-17-0605

[33] Goodman RH , Smolik S . CBP/p300 in cell growth, transformation, and development. Genes Dev. 2000; 14(13): 1553‐1577. 10887150

[34] Nguyen DX , Chiang AC , Zhang XH , et al. WNT/TCF signaling through LEF1 and HOXB9 mediates lung adenocarcinoma metastasis. Cell. 2009; 138(1): 51‐62. 19576624 10.1016/j.cell.2009.04.030 PMC2742946

[35] Xie P , Yuan FQ , Huang MS , et al. DCBLD2 affects the development of colorectal cancer via EMT and angiogenesis and modulates 5‐FU drug resistance. Front Cell Dev Biol. 2021; 9: 669285. 34095137 10.3389/fcell.2021.669285 PMC8170045

[36] Kamposioras K , Vassilakopoulou M , Anthoney A , et al. Prognostic significance and therapeutic implications of Caveolin‐1 in gastrointestinal tract malignancies. Pharmacol Ther. 2021;233:108028. 34755606 10.1016/j.pharmthera.2021.108028

[37] Takemasa I , Yamamoto H , Sekimoto M , et al. Overexpression of CDC25B phosphatase as a novel marker of poor prognosis of human colorectal carcinoma. Cancer Res. 2000; 60(11): 3043‐3050. 10850455

[38] Makondi PT , Chu CM , Wei PL , et al. Prediction of novel target genes and pathways involved in irinotecan‐resistant colorectal cancer. PLoS One. 2017; 12(7): e0180616. 28749961 10.1371/journal.pone.0180616 PMC5531462

[39] Montorsi L , Guizzetti F , Alecci C , et al. Loss of ZFP36 expression in colorectal cancer correlates to wnt/ß‐catenin activity and enhances epithelial‐to‐mesenchymal transition through upregulation of ZEB1, SOX9 and MACC1. Oncotarget. 2016; 7(37): 59144‐59157. 27463018 10.18632/oncotarget.10828 PMC5312301

[40] Li Y , Zhang H , Li Q , et al. CDK12/13 inhibition induces immunogenic cell death and enhances anti‐PD‐1 anticancer activity in breast cancer. Cancer Lett. 2020; 495: 12‐21. 32941949 10.1016/j.canlet.2020.09.011

[41] Yokoyama Y , Zhu H , Lee JH , et al. BET inhibitors suppress ALDH activity by targeting ALDH1A1 super‐enhancer in ovarian cancer. Cancer Res. 2016; 76(21): 6320‐6330. 27803105 10.1158/0008-5472.CAN-16-0854 PMC5117661

[42] Zhang J , Liu W , Zou C , et al. Targeting super‐enhancer‐associated oncogenes in osteosarcoma with THZ2, a covalent CDK7 inhibitor. Clin Cancer Res. 2020; 26(11): 2681‐2692. 31937612 10.1158/1078-0432.CCR-19-1418

[43] Ye B , Fan D , Xiong W , et al. Oncogenic enhancers drive esophageal squamous cell carcinogenesis and metastasis. Nat Commun. 2021; 12(1): 4457. 34294701 10.1038/s41467-021-24813-2 PMC8298514

[44] Dong J , Li J , Li Y , Ma Z , Yu Y , Wang C‐Y . Transcriptional super‐enhancers control cancer stemness and metastasis genes in squamous cell carcinoma. Nat Commun. 2021;12(1). 10.1038/s41467-021-24137-1 PMC8233332 34172737

[45] Jin Y , Zhang P , Wang Y , et al. Neddylation blockade diminishes hepatic metastasis by dampening cancer stem‐like cells and angiogenesis in uveal melanoma. Clin Cancer Res. 2018; 24(15): 3741‐3754. 29233905 10.1158/1078-0432.CCR-17-1703

[46] Guo L , Li B , Lu Z , et al. CCDC137 is a prognostic biomarker and correlates with immunosuppressive tumor microenvironment based on pan‐cancer analysis. Front Mol Biosci. 2021; 8: 674863. 34055889 10.3389/fmolb.2021.674863 PMC8155610

[47] Charbord P , Pouget C , Binder H , et al. A systems biology approach for defining the molecular framework of the hematopoietic stem cell niche. Cell Stem Cell. 2014; 15(3): 376‐391. 25042701 10.1016/j.stem.2014.06.005

[48] Wang WD , Wu GY , Bai KH , et al. A prognostic stemness biomarker CCDC80 reveals acquired drug resistance and immune infiltration in colorectal cancer. Clin Transl Med. 2020; 10(6): e225. 33135356 10.1002/ctm2.225 PMC7603297

[49] Yang L , Liu Q , Zhang X , et al. DNA of neutrophil extracellular traps promotes cancer metastasis via CCDC25. Nature. 2020;583(7814):133‐138. 32528174 10.1038/s41586-020-2394-6

[50] Lui GYL , Grandori C , Kemp CJ . CDK12: an emerging therapeutic target for cancer. J Clin Pathol. 2018;71(11):957‐962. 30104286 10.1136/jclinpath-2018-205356 PMC6242340

