# Supplementary material for: CDK12 orchestrates super‐enhancer‐associated CCDC137 transcription to direct hepatic metastasis in colorectal cancer
Source: Clin Transl Med. 2022 Oct 17;12(10):e1087. doi: 10.1002/ctm2.1087 (PMC9577262; doi:10.1002/ctm2.1087)
Supplement: Supplementary file 1 — Supporting Information [file CTM2-12-e1087-s001.docx]

Supporting Information

**CDK12 orchestrates super-enhancer-associated CCDC137 transcription to direct hepatic metastasis in colorectal cancer**

Wei Dai^1†^, Junhong Wu^1†^, Xiaopeng Peng^1^, Wen Hou^1^, Hao Huang^1^, Qilai Cheng^1^, Zhiping Liu^2^, Walter Luyten^3^, Liliane Schoofs^3^, Jingfeng Zhou^4*^, Shenglan Liu^1*^

^1^School of Pharmacy, Gannan Medical University, Ganzhou 341000, China.

^2^Center for Immunology, Gannan Medical University, Ganzhou 341000, China.

^3^Department of Biology, KU Leuven, Naamsestraat 593000, Leuven, Belgium. ^4^Department of Hematology and Oncology, International Cancer Center, Shenzhen Key Laboratory, Shenzhen University General Hospital, Shenzhen University Clinical Medical Academy, Shenzhen University Health Science Center, Xueyuan AVE 1098, Shenzhen 518000, China.

†These authors contributed equally to this work.

***Corresponding Authors:**

Shenglan Liu, School of Pharmacy, Gannan Medical University, Ganzhou, Jiangxi, 341000, China, Phone: +86-0797-8169758, Email: liushl5@gmu.edu.cn

Or Jingfeng Zhou, Shenzhen University General Hospital, Shenzhen 518000, China, Email: jingfengzhou@foxmail.com

**This Word file includes:**

Supplementary Materials and Methods

Figure. S1 to S8

Tables S1 to S5

**Funding information**

National Natural Science Foundation of China, Grant/Award Numbers: 82003797, 82260718, 82003801, 81903635 and 82270172; NSFC Projects of International Cooperation and Exchanges Grant/Award Numbers: 82111530101; Shenzhen Science and Technology Program, Grant/Award Numbers: RCBS20210706092216031; Shenzhen Key Laboratory Foundation Grant/Award Numbers: ZDSYS20200811143757022.

**Supplementary Materials and Methods**

**Cell culture**

Human normal colonic epithelial cells including NCM460 and HCoEpiC cultured in MEM medium supplemented with 10% fetal bovine serum (FBS, Gibco, NY, USA) with 1% penicillin-streptomycin (Solarbio, Beijing, China) were purchased from Incell Corporation (San Antonio, TX) and ScienCell (Carlsbad, CA), respectively. The human CRC cell lines HCT116, HCT8, COLO205 and MC38 were obtained from the American Type Culture Collection (ATCC, Manassas, VA) and grown in RPMI 1640 supplemented with 10% FBS. 293T and SW620 cells from ATCC were cultured in DMEM medium containing 10% FBS. All the cell lines were tested and authenticated by using short tandem repeat (STR) matching analysis.

**Generating CRC Sublines with highly liver-metastatic competence**

We intrasplenically injected 5×10^5^ MC38 cells stably expressing luciferase (MC38-luc) into C57BL/6 mice. Twelve days later, a single nodule on liver surface was picked up to expand culture, which referred liver metastatic derivative 1 (LM1). The MC38-LM1 cells were then reinoculated into the secondary receipt C57BL/6 mice, producing MC38-LM2 cells. The MC38-LM2 cells were subjected to the third round of *in vivo* selection, yielding ML38-LM3 sublines, which were confirmed to own extremely increased capacity of liver metastasis.

**Cell viability assay**

Cell viability was measured using CCK8 assay (TargetMol, Shanghai, China). Briefly, cells were seeded into a 96-well plate overnight at a density of 5000 cells per well. Cells were treated with increasing concentrations of SR-4835 for 68 h, followed by CCK8 (10 μL/well) treatment for another 4 h. The optical density was read with a wave length of 450 nm. Cell viability was calculated using the following formula: Relative viability = (Mean absorbance of treated wells−background absorbance) / (Mean absorbance of untreated wells−background absorbance) × 100%. The half-maximal inhibitory concentration (IC_50_) of SR-4835 was determined via curve fitting of the sigmoidal dose-response curve (GraphPad Prism 5, La Jolla, CA).

**Cell growth curve**

CRC cells were seeded into 24-well plates at a density of 1×10^4^ per well and allowed growing for 6 days. Cell numbers of triple wells in each group were counted with trypan blue staining every day.

**Cell migration and invasion assays**

After exposure to SR-4835 for 24 h, HCT116 and SW620 cells were harvested, resuspended in serum-free medium and seeded into 24-well transwell inserts with 8 μm pore size (Corning, NY, USA). For migration assay, inserts were not coated with matrigel. For invasion assay, inserts were pre-coated with 30% matrigel (Corning, NY, USA). After incubation for 24 h, migrated or invaded cells were fixed with 4% paraformaldehyde and stained in 0.1% crystal violet. Migrated or invaded cells of 3 random fields were counted and photographed with an inverted microscope.

**Aldehyde dehydrogenase assay**

Aldehyde dehydrogenase (ALDH) assay was performed with the ALDEFLUOR^TM^ kit (Stem Cell Technologies, Vancouver, BC, Canada) following to the manufacturer’s instructions. HCT116 and SW620 cells were treated with 100 nM SR-4835 for 48 h and suspended in 500 μL ALDH assay buffer containing ALDH reagent with or without the DEAB reagent at 37 °C for 45 min. Cells were then washed, resuspended in ALDH assay buffer and subjected to analysis with flow cytometer.

**Western blotting assay**

CRC cells or tissues were lysed in RIPA lysis buffer containing 1×PBS, 0.5% sodium deoxycholate, 1% NP-40, and 0.1% SDS. Equal protein samples (30 μg) were separated by SDS-PAGE gel electrophoresis and transferred to nitrocellulose membranes, followed by incubation with the primary antibodies at 4 ℃ overnight. The β-actin was used as the loading control and protein bands were visualized by ECL chemiluminescence (Millipore, Danvers, USA).

**Apoptosis assay by flow cytometry**

After CRC cells were treated with increasing concentrations of SR-4835 for 24 h (HCT8 and COLO205) or 48 h (HCT116 and SW620), cells were collected for staining with 0.3 μL Annexin V-FITC (Sigma-Aldrich, Shanghai) for 30 minutes in the dark, followed by apoptosis evaluated by flow cytometer analysis (BD FACSLSR Fortassa) immediately after staining with 1 μL PI.


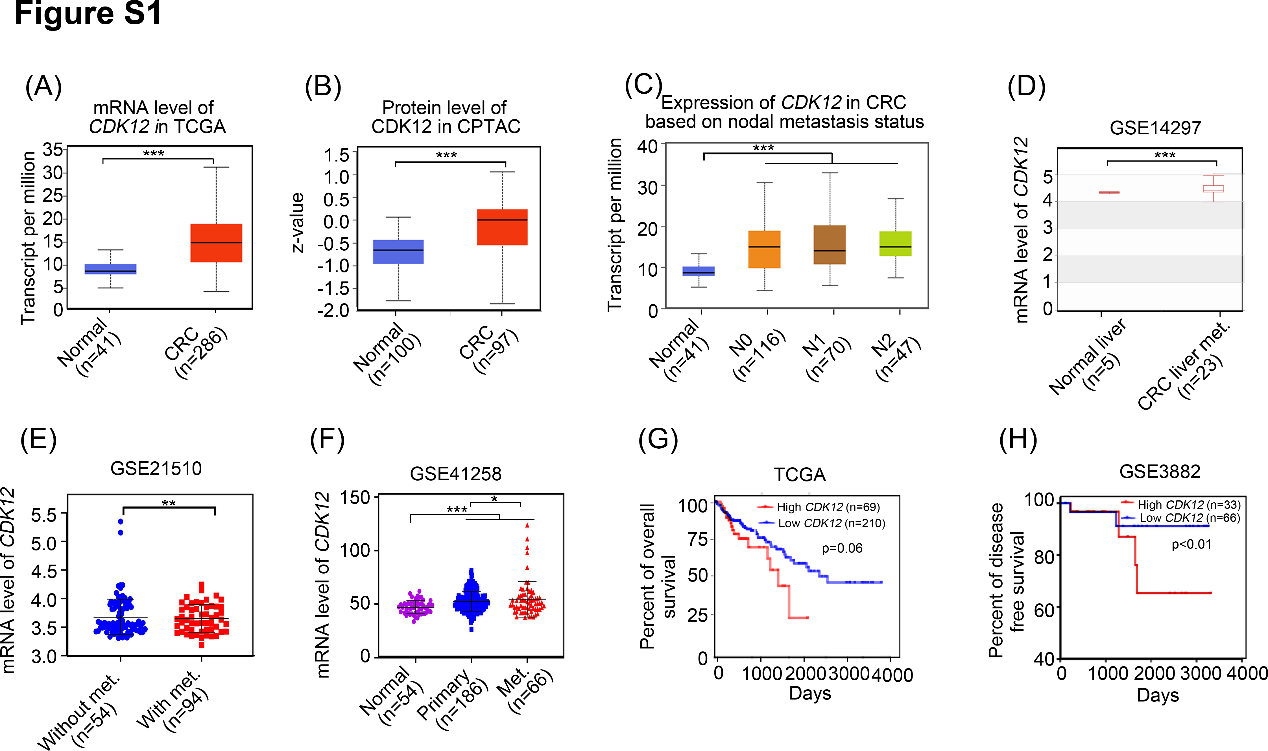


**Figure. S1** CDK12 is overexpressed and correlated with poor prognosis in patients with CRC. (A-B) The mRNA (A) and protein (B) levels of CDK12 were shown in patients with CRC and normal adjacent tissues obtained from TCGA and CPTAC database respectively. (C) The transcriptional levels of CDK12 were grouped by lymph node metastasis stages of CRC in TCGA database. (D) The microarray expression data for CDK12 in normal liver tissues and CRC liver metastatic tissues was obtained from the Human Cancer Metastasis Database. (E) The mRNA levels of CDK12 were presented in CRC patients with metastasis or without metastasis in GSE21510. (F) The mRNA levels of CDK12 were presented in human primary CRC tumors, liver metastatic CRC tumors and adjacent normal tissue in GSE41258. (G-H) Overall survival (G) and disease-free survival (H) based on CDK12 expression in CRC patients in the cohort of TCGA and GEO database were shown, respectively, log-rank test. ***, *P<*0.001, Student’s *t* test for results in (A,B) and (D,E). *, *P<*0.05; ***, *P<*0.001, one-way ANOVA with *post hoc* intergroup comparison by Tukey's test for results in (C) and (F). Log-rank test for results in (G) and (H).


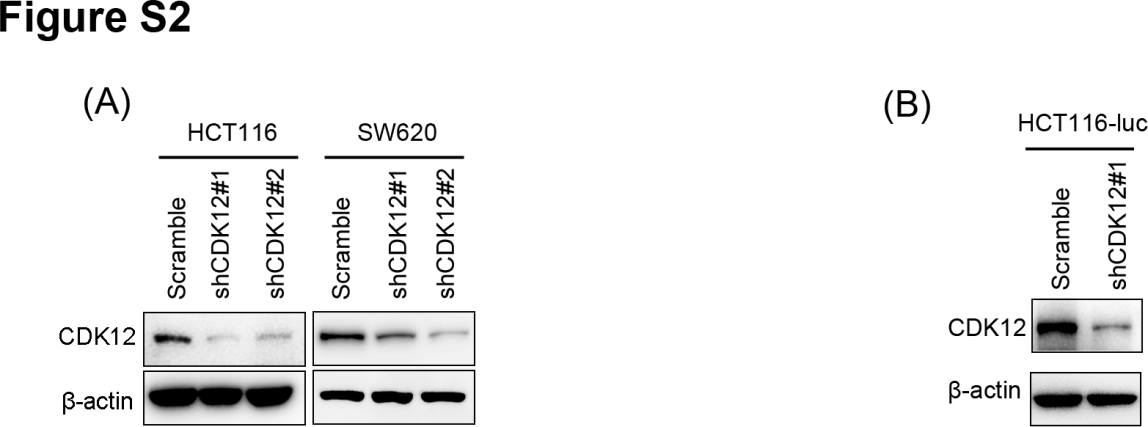


**Figure. S2** The knockdown efficiency of CDK12 in CRC cells was examined by Western blotting assay. (A) HCT116 and SW620 cells were transduced with lentiviral shRNA against CDK12, and then incubated in 1 μg/mL puromycin for 5 days to select stable clones, followed by Western blotting analysis. (B) HCT116-luc were transduced with lentiviral shRNA against CDK12, and then incubated in 1 μg/mL puromycin for 5 days to select stable clones, followed by Western blotting analysis.


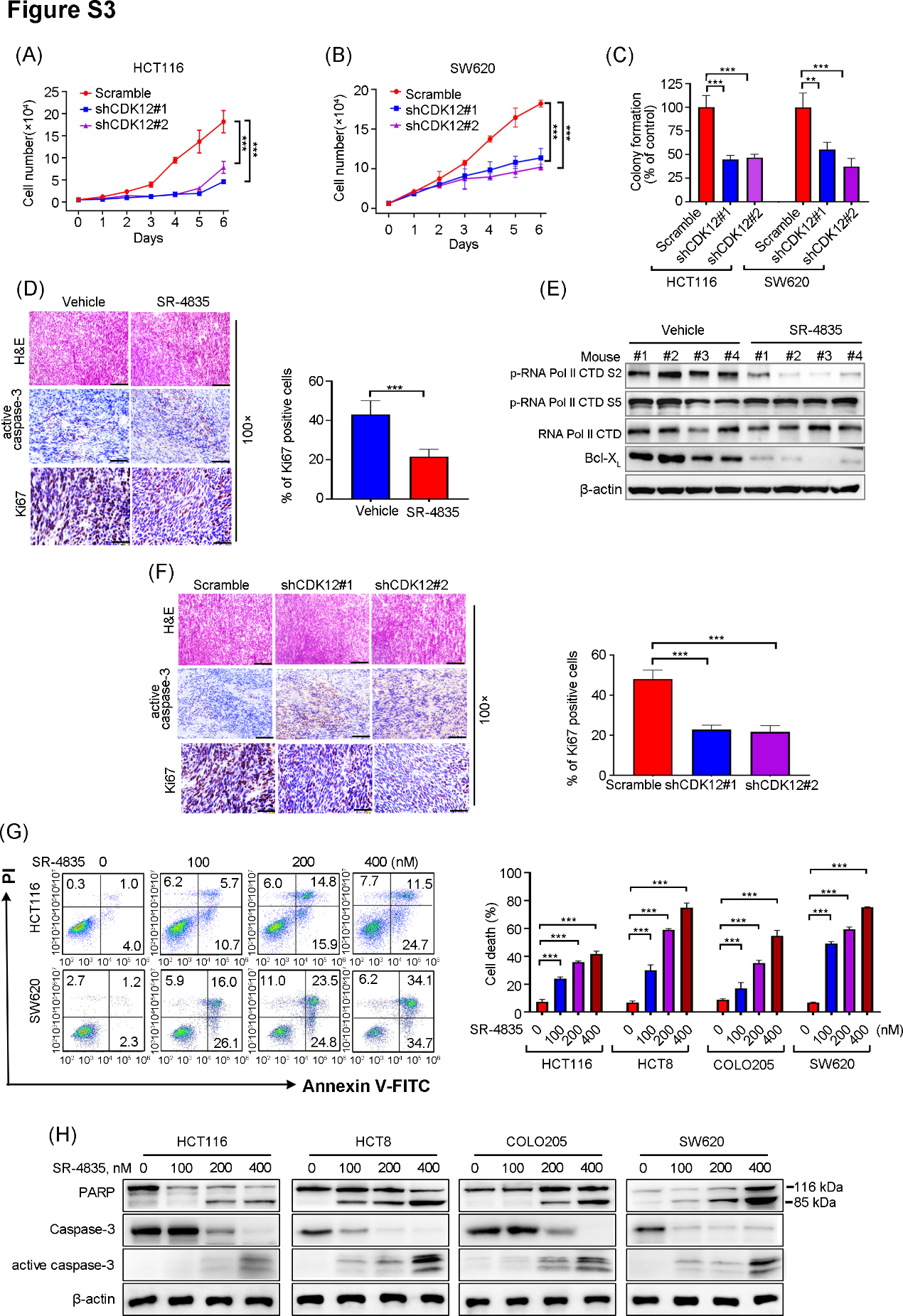


**Figure S3** CDK12 intervention inhibits proliferation and survival in CRC. (A-B) CDK12-silenced HCT116 (A) and SW620 cells (B) were daily subjected to trypan blue exclusion assay, respectively. (C) Colony formation abilities were detected in CDK12-depleted HCT116 and SW620 cells. (D) *Left*, Representative images of H&E and IHC staining with active caspase-3 or Ki67 in tumor tissues from mice treated with vehicle or SR-4835. Scale bar, 100 μm. *Right*, The percent of Ki67-positive cells in tumor sections was counted. n=3 per condition. (E) The CTD phosphorylation of RNA Pol II in the tumors from the indicated xenografts was analyzed with Western blotting assay. (F) *Left*, Representative images of H&E and IHC staining with active caspase-3 or Ki67 in tumor sections from mice inoculated with Scramble or CDK12-silenced HCT116 cells were shown. Scale bar, 100 μm. *Right*, The percent of Ki67-positive cells in indicated tumor sections was counted. n=3 per condition. (G) CRC cells were incubated with increasing concentrations of SR-4835, and then subjected to flow cytometry analysis after Annexin V-FITC/PI dual staining. Representative flow dot plots *(left)* and quantitative analysis from 3 independent experiments *(right)* were shown. (H) The cleavage of PARP and active caspase-3 in SR-4835-treated CRC cells were analyzed with Western blotting assay. **, *P*<0.01; ***, *P*<0.001, one-way ANOVA with *post hoc* intergroup comparison by Tukey's test for results in (A-C)**,** (F) and (G). ***, *P*<0.001, Student’s *t* test for results in (D).


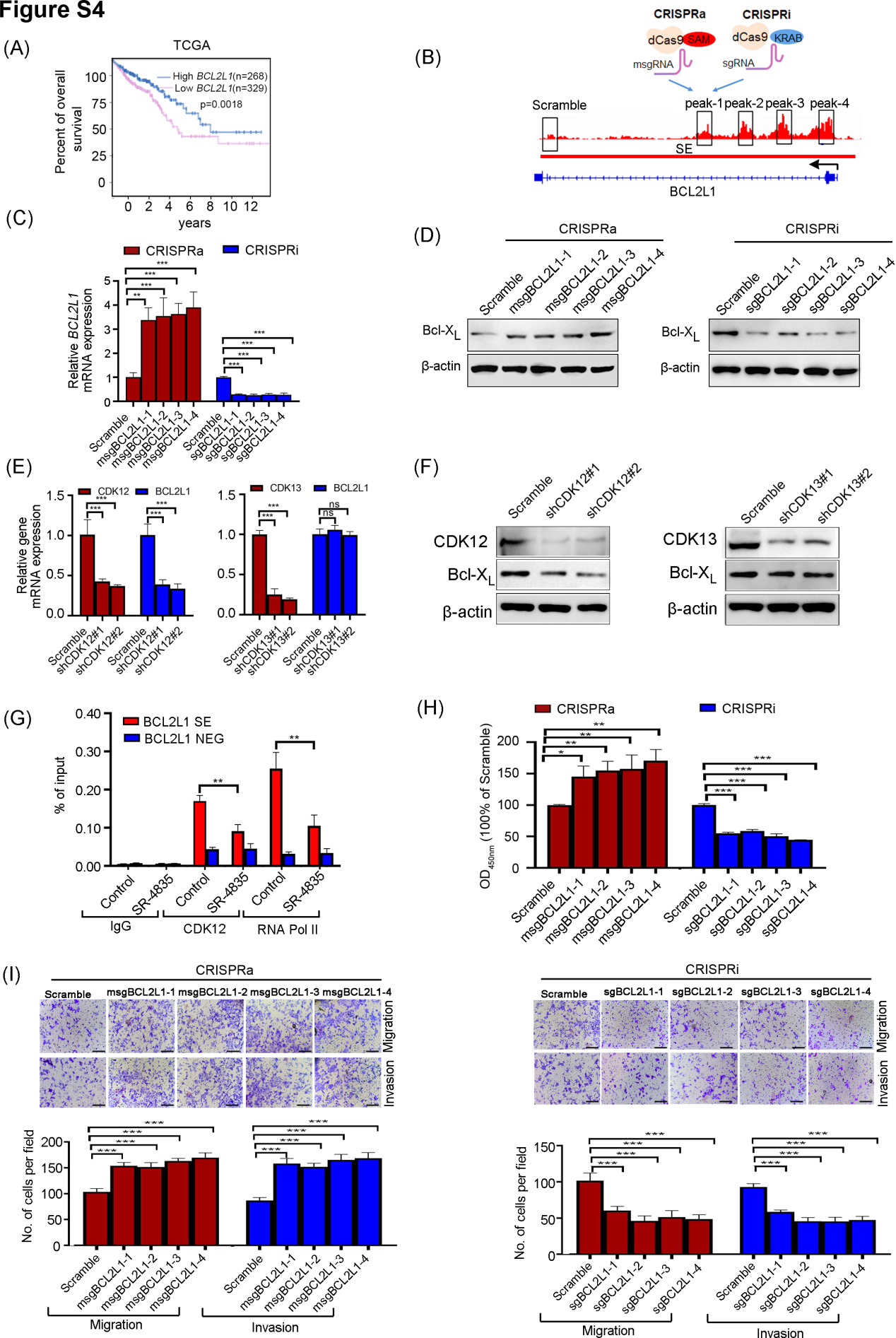


**Figure S4** *BCL2L1* is the SE-associated gene regulated by CDK12 in CRC. (A) Overall survival of CRC patients based on the transcription levels of *BCL2L1* in the cohort of TCGA database was shown. (B) The schematic shows targeting SE peaks of BCL2L1 with four individual gRNAs (gBCL2L1-1 to gBCL2L1-4) utilizing a CRISPRa system with the transcriptional activator (SAM)-associated catalytically dead Cas9 (dCas9) or a CRISPRi system with the repressor complex (KRAB-MeCP2)-associated dCas9. (C) The qRT-PCR results showed the mRNA levels of *BCL2L1* were elevated or reduced by employing the CRISPRa or CRISPRi system targeting individual SE peaks of *BCL2L1* in HCT116 cells. (D) Western blotting showed the protein levels of Bcl-X_L_ were upregulated or downregulated by introducing the CRISPRa or CRISPRi system targeting of *BCL2L1* individual SE peaks in HCT116 cells. (E) Knockdown of CDK12 but not CDK13 inhibited the mRNA levels of *BCL2L1* in HCT116 cells. (F) Knockdown of CDK12 but not CDK13 inhibited the protein levels of Bcl-X_L_ in HCT116 cells. (G) ChIP-qPCR results showed that treatment of SR-4835 decreased the occupancy of CDK12 and RNA pol II on the SE region of *BCL2L1* with no obvious alteration on the negative control region (NEG) in HCT116 cells. (H) The CCK8 assay showed the rates of proliferation were enhanced or inhibited in HCT116 cells by employing the CRISPRa or CRISPRi system targeting of *BCL2L1* individual SE peaks. (I) The transwell assay showed the abilities of migration and invasion were increased or decreased in HCT116 cells by employing the CRISPRa *(left)* or CRISPRi *(right)* system targeting of *BCL2L1* individual SE peaks. Scale bar: 200 μm. Log-rank test for results in (A). **, *P*<0.01; ***, *P*<0.001, one-way ANOVA with *post hoc* intergroup comparison by Tukey's test for results in (C)**,** (E)**,** (H) and (I)**.** **, *P*<0. 01, Student’s *t* test for results in (G).


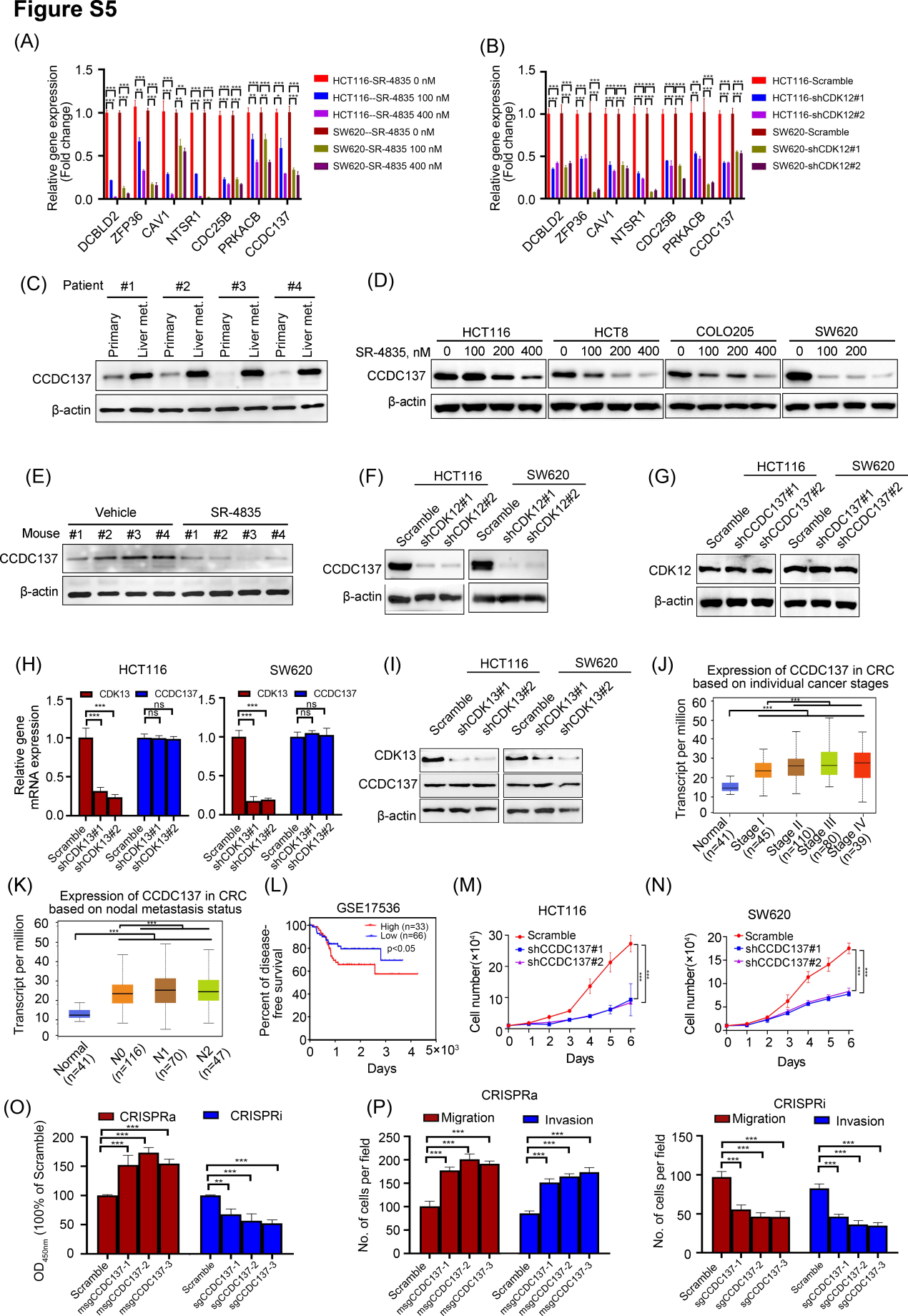


**Figure S5** CCDC137 is an SE-associated gene regulated by CDK12 in CRC cells. (A) qRT-PCR revealed that the transcriptional levels of candidate genes were significantly reduced after exposure to indicated concentrations of SR-4835 for 6 h in HCT116 and SW620 cells. (B) qRT-PCR revealed that the mRNA levels of candidate genes were significantly decreased in CDK12-depleted HCT116 and SW620 cells. (C) The protein levels of CCDC137 in primary CRC tissues and liver metastatic CRC tissues were examined by Western blotting assay. (D) After treated with SR-4835 for 24 or 48 h, CRC cells were collected and subjected to Western blotting analysis. (E) Western blotting analysis of CCDC137 expression in tumor sections from mice treated with vehicle or SR-4835. (F) Western blotting assay showed that the expression of CCDC137 in CDK12-silenced CRC cells. (G) Western blotting analysis showed that CDK12 expression in CCDC137-depleted CRC cells. (H) Knockdown of CDK13 had no effect on the mRNA levels of *CCDC137* in CRC cells. (I) CDK13 knockdown had no effect on the protein levels of CCDC137 in CRC cells. (J) Expression of CCDC137 in CRC based on individual cancer stages was obtained from TCGA database. (K) Expression of CCDC137 in CRC based on nodal metastasis status was obtained from TCGA database. (L) Disease-free survival based on CDK12 expression in CRC patients in the cohort of GSE17536. (M-N) CDK12-silenced HCT116 (M) and SW620 cells (N) were subjected to trypan blue exclusion assay, respectively. (O) The CCK8 assay showed the rates of proliferation were enhanced or inhibited by employing the CRISPRa or CRISPRi system targeting of *CCDC137* individual SE peaks in HCT116 cells. (P) The transwell assay showed the abilities of migration and invasion were enhanced or inhibited by employing the CRISPR*a (left)* or CRISPRi *(right)* system targeting of *CCDC137* individual SE peaks in HCT116 cells. ns, not significant; *, *P<*0.05; **, *P<*0.01; ***, *P*<0.001, one-way ANOVA with *post hoc* intergroup comparison by Tukey's test for results in (A,B), (H), (J-K) and (M-P). Log-rank test for results in (L).


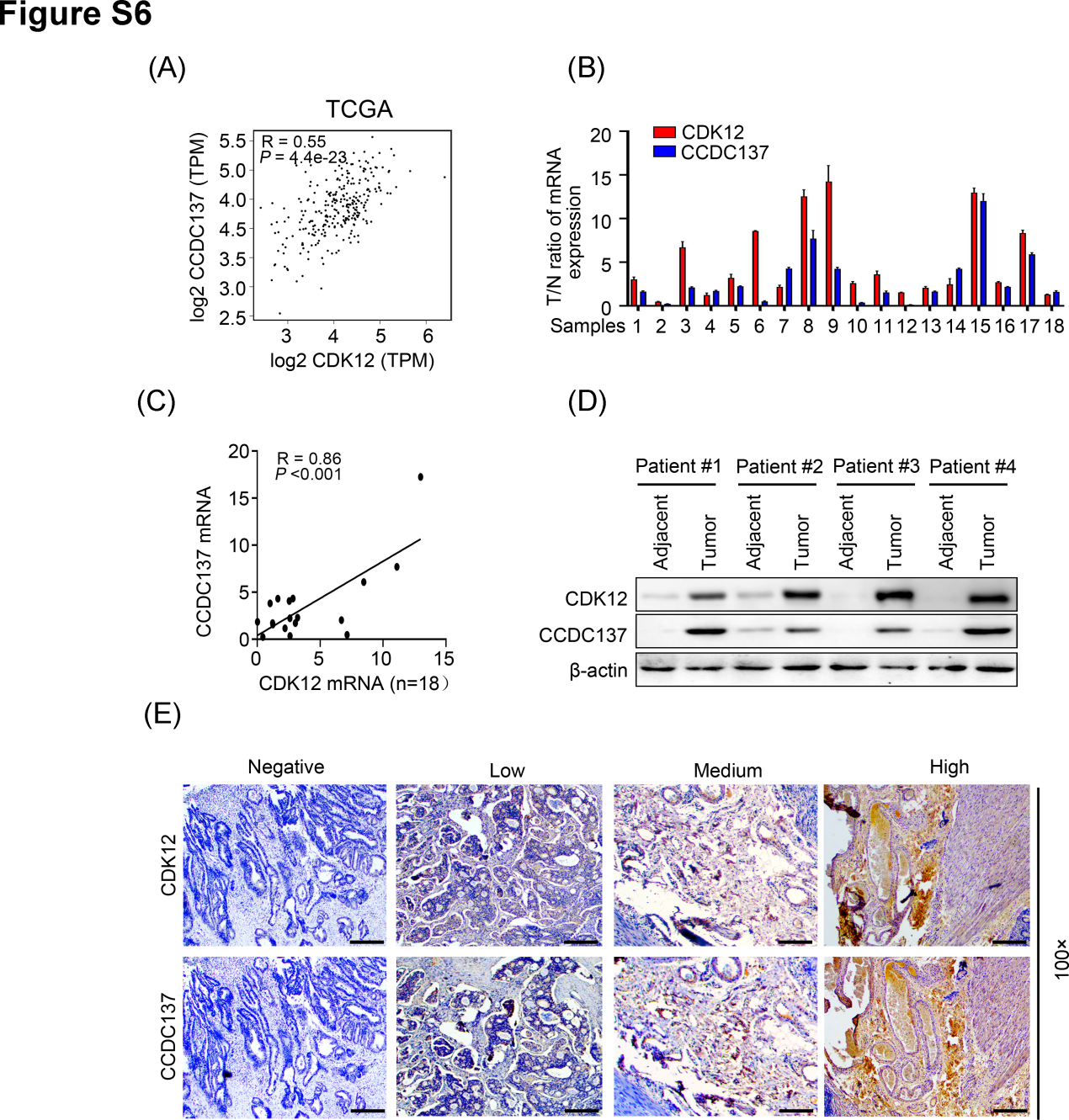


**Figure S6** CDK12 expression level positively correlates with CCDC137 expression in CRC patients. (A) The correlation of CDK12 and CCDC137 in CRC patients from TCGA database was analyzed. (B) Average tumor/adjacent normal intestine epithelium (N/T) ratios of CDK12 and CCDC137 expression were quantified by qRT-PCR and normalized against GAPDH (n=18), results were calculated from three parallel experiments. (C) The relationship between CDK12 and CCDC137 mRNA expression in the tumor samples described in (B) was analyzed by Spearman’s correlation (n=18). (D) The CDK12 and CCDC137 protein expression in the freshly collected CRC samples were examined by Western blotting assay. (E) IHC results in CRC tissues showed that CDK12 expression was positively correlated with CCDC137 expression, Scale bar, 200 µm. Spearman’s correlation analysis was used to for results in (A) and (C).


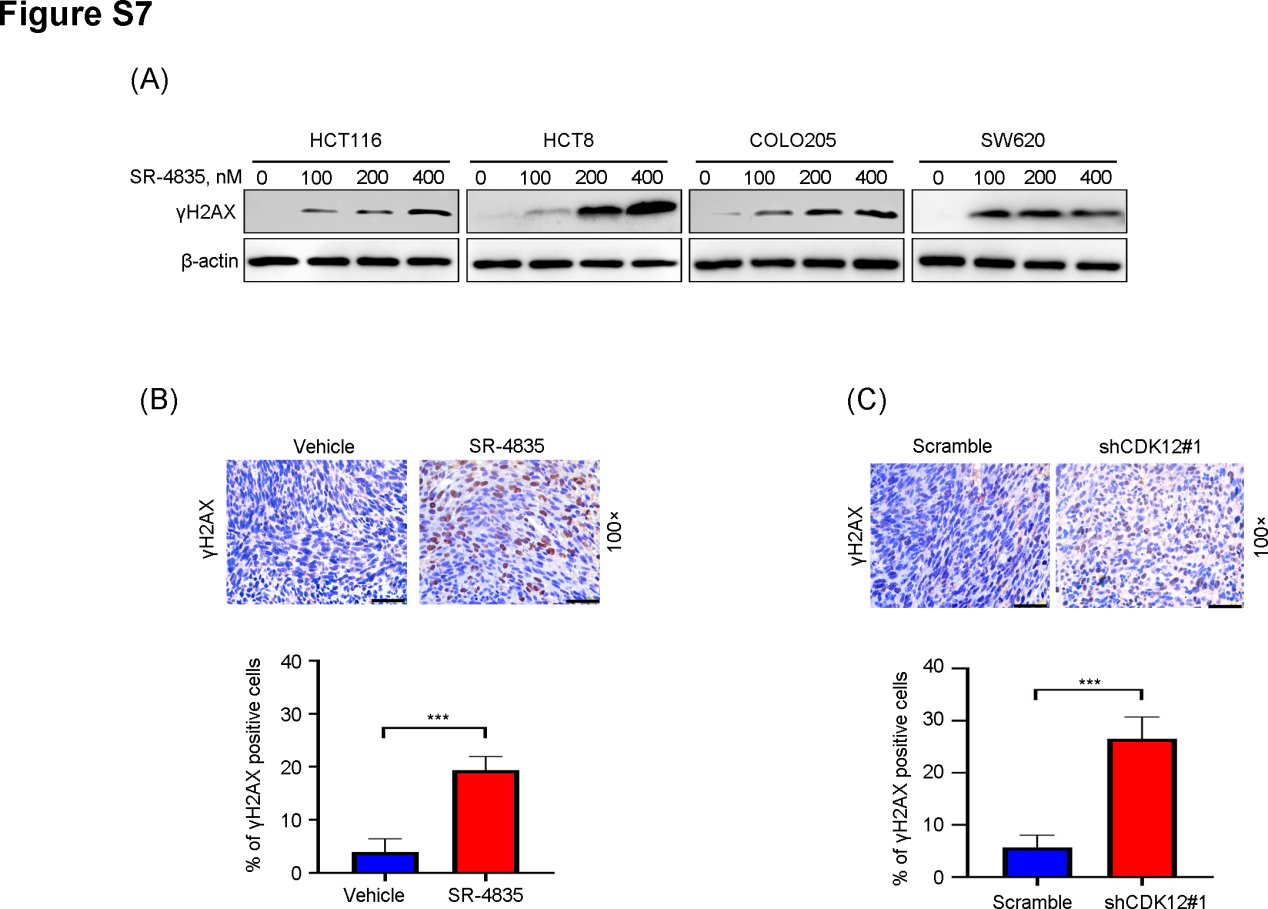


**Figure S7** CDK12 inhibition induces DNA damage in CRC cells. (A) After SR-4835 treated with 24 or 48 h. CRC cells were subjected to Western blotting analysis. (B) The percent of γH2AX-positive cells was counted in tumors from vehicle-treated or SR-4835-treated xenografts. n=3 per condition. Scale bar, 200 µm. (C) The percent of γH2AX-positive cells was counted in xenografts from Scramble or CDK12-konckdown group. n=3 per condition. Scale bar, 200 µm. **, *P*<0.01; ***, *P<*0.001, Student’s *t* test.


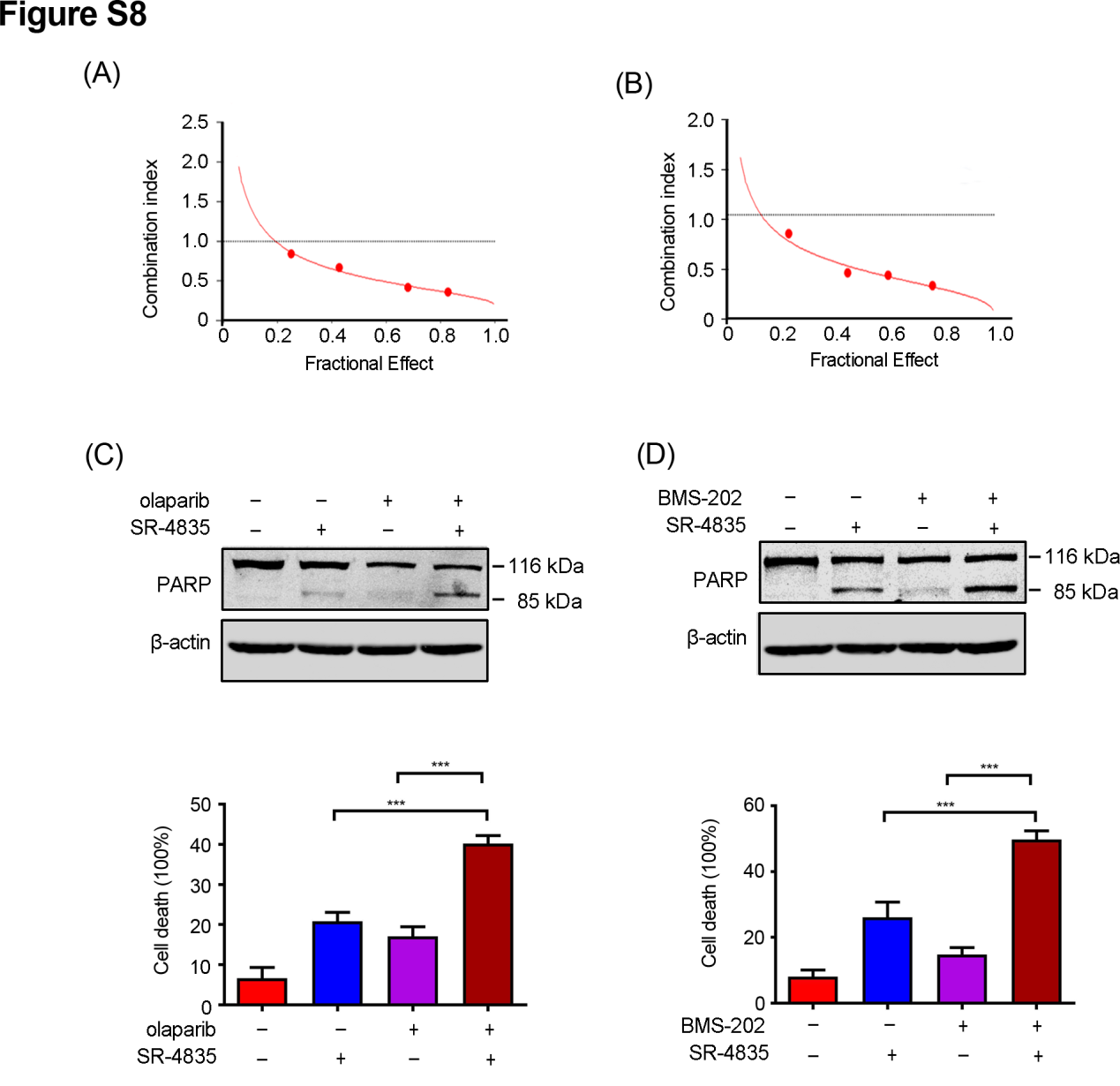


**Figure S8.** SR-4835 is synergistic with the PARP inhibitors (olaparib) and PD-L1 inhibitor (BMS-202). (A-B) HCT116 cells were incubated with a serially fixed ratio combining SR-4835 and olaparib (A) or BMS-202 (B) for 72 h. The synergistic effect of these two agents was calculated according to the method of Chou and Talalay. The combination index (CI) < 1 indicates a synergetic effect. (C) HCT116 cells were treated with SR-4835 (100 nM) and olaparib (2.5 μM) for 48 h, followed by Western blotting *(Top)* and trypan blue assay *(Bottom).* (D) HCT116 cells were treated with SR-4835 (100 nM) and BMS-202 (2 μM) for 48 h, followed by Western blotting *(Top)* and trypan blue assay *(Bottom).* ***, *P*<0.001, one-way ANOVA with post-hoc intergroup comparison with Tukey’s test for results in (C,D).

**Supplementary Table S1. Primers for qRT-PCR**

| Gene | Forward | Reverse |
| --- | --- | --- |
| *CDK7* | 5'-ATGGCTCTGGACGTGAAGTC-3' | 5'-CTTAATGGCGACAATTTGGTTG-3' |
| *CDK12* | 5'-GGAGTCCACTCCCCAGTAGG-3' | 5'-TGGAATGACGACTGCGTGAA-3' |
| *CDK13* | 5'-GCTGACCATAAGCAGCTAACCT-3' | 5'-CCAGCCACTCTCCCAACATAA-3' |
| *BRD4* | 5'-AAGCCAAAGACCCGTGTAGG-3' | 5'-CTCCGCAGACATGCTAGTGA-3' |
| *EP300* | 5'-GCAGTGTGCCAAACCAGATG-3' | 5'CATAGCCCATAGGCGGGTTG-3' |
| *MED1* | 5'-AGCACCCTTCTCTTCTTGCC-3' | 5'-GGAGAAGCTAGATCCGCCAC-3' |
| *BCL2L1* | 5'-CCTGCCTGCCTTTGCCTAA-3' | 5'-CCCGGTTGCTCTGAGACATT-3' |
| *DCBLD2* | 5'-ACCTCGGCATGTACTGTAGTC-3' | 5'-GATACCTGTTTCCTTGTCCTGA-3' |
| *NTSR1* | 5'- CCCCATCCTGTACAACCTCG-3' | 5'-CTAGTACAGCGTCTCGCGG-3' |
| *CCDC137* | 5'-GAGCAGCGGTGTCCAGG-3' | 5'-TCTTCTTCTCTTTGCTGCGA-3' |
| *CDC25B* | 5'-TCGGCTCTCAGTCCAGCA-3' | 5'-ACTCTTTGGGGTTTCGCTGC-3' |
| *PRKACB* | 5'-TCCACAGCTAGCAGTAAGAGC-3' | 5'-TTGCGTGACAATCCCATGTTG-3' |
| *ZFP36* | 5'-GAAGGGCCACTCCTATCAGC-3' | 5'- AAAACTCCCGCCTCGAAGAC-3' |
| *CAV1* | 5'-TGTCCGCTTCTGCTATCTGC-3' | 5'-TAGGCCCCCTCTCCATTAGG-3' |
| *GAPDH* | 5'-GCAAATTCCATGGCACCGTC-3' | 5'-TCGCCCCACTTGATTTTGG-3' |

**Supplementary Table S2. The target sequence of gRNAs.**

| gRNA | Sequence |
| --- | --- |
| *gBCL2L1 Scramble* | TTGCCCAGGCTGGAGTGCAA |
| *gBCL2L1-1* | CCTTTGGTACCCCATGGCAG |
| *gBCL2L1-2* | CATTCTAGGCTGCCATGAAC |
| *gBCL2L1-3* | CTTGAAGGGATGATCACAGC |
| *gBCL2L1-4* | ATGCAAAATGAGAGAGGGGG |
| *gCCDC137 Scramble*  *gCCDC137-1* | TTGCCCAGGCTGGAGTGCAA  TCACCGCAACCTCCACCTCT |
| *gCCDC137-2*  *gCCDC137-3* | TCACAAAGCCCAGGCCTCTC  CCCTCCTCTGTCTGTGCCCG |

**Supplementary Table S3. The sequence of shRNA.**

| shRNA | Sequence |
| --- | --- |
| *pLKO.1-non-target shRNA* | CCGGGCGCGATAGCGCTAATAATTTCTCGAGAAATTATTAGCGCTATCGCGCTTTTT |
| *Human shCDK12#1* | CCGGCCCGCAAAGAGCGAGAGTTAACTCGAGTTAACTCTCGCTCTTTGCGGGTTTTTG |
| *Human shCDK12#2* | CCGGCAGTCAAGAAAGCCCTAATAACTCGAGTTATTAGGGCTTTCTTGACTGTTTTTG |
| *Human shBCL2L1#1* | CCGGATGGTTATCTTACGACTGTTACTCGAGTAACAGTCGTAAGATAACCATTTTTTG |
| *Human sh BCL2L1#2* | CCGGCAGGAGAACCACTACATGCAACTCGAGTTGCATGTAGTGGTTCTCCTG TTTTTG |
| *Human shCCDC137#1* | CCGGCTAGATAAAGTCCGACGGAAACTCGAGTTTCCGTCGGACTTTATCTAG TTTTTG |
| *Human shCCDC137#2*  *Human shCDK13#1*  *Human shCDK13#2* | CCGGACCAGGACGAACAGGAGATTCCTCGAGGAATCTCCTGTTCGTCCTGGT TTTTTG  CCGGAGTGTTATTGTGAAAGGTGTACTCGAGTACACCTTTCACAATAACACT TTTTTG  CCGGCGATGTCTTCTTGCTGATTTACTCGAGTAAATCAGCAAGAAGACATCGTTTTTG |

**Supplementary Table S4. Primers for ChIP-qPCR**

| Gene | Forward | Reverse |
| --- | --- | --- |
| *BCL2L1* NEG | 5'-TCTGTCTCAAAGAAAACCTCTACA -3' | 5'-GGCACAGATATATAGATCCTCCACT-3' |
| *BCL2L1* SE | 5'-AGAAAGGATACAGCTGGAGTCAG-3' | 5'- CATTGATGGCACTGGGGGT-3' |
| *CCDC137* NEG | 5'- ACGGTGAAGTTTGGTGAGGT-3' | 5'-ACAAACTCAAGTTGTCCCCGT -3' |
| *CCDC137* SE | 5'-CTTTGTGGTTCTGGACGAGGT-3' | 5'- CTCACTCAGTGGCTGTCCCT-3' |

**Supplementary Table S5. Limiting dilution analysis in BALB/c nude mice.**

|  | **Engrafted mice** |  |
| --- | --- | --- |
| Cell number  5×10^6^  1×10^6^  5×10^5^  1×10^5^  Frequency | Vehicle  5/5  4/5  2/5  0/5  1/701,405 | SR4835  3/5  1/5  0/5  0/5  1/4,099,078 |
